# Low‐Temperature Lithium Metal Batteries Achieved by Synergistically Enhanced Screening Li^+^ Desolvation Kinetics

**DOI:** 10.1002/adma.202411601

**Published:** 2024-12-16

**Authors:** Fengyi Zhu, Jian Wang, Yongzheng Zhang, Haifeng Tu, Xueqing Xia, Jing Zhang, Haiyan He, Hongzhen Lin, Meinan Liu

**Affiliations:** ^1^ State Key Laboratory of Featured Metal Materials and Life‐cycle Safety for Composite Structures Guangxi Key Laboratory of Processing for Non‐Ferrous Metals and Featured Materials School of Resources Environment and Materials Guangxi University Nanning 530004 China; ^2^ i‐lab & CAS Key Laboratory of Nanophotonic Materials and Devices Suzhou Institute of Nano‐Tech and Nano‐Bionics Chinese Academy of Sciences Suzhou 215123 China; ^3^ College of Mechanics and Materials Hohai University Nanjing 210098 China; ^4^ Helmholtz Institute Ulm (HIU) 89081 Ulm Germany; ^5^ Karlsruhe Institute of Technology (KIT) D‐76021 Karlsruhe Germany; ^6^ State Key Laboratory of Chemical Engineering East China University of Science and Technology Shanghai 200237 China; ^7^ School of Materials Science and Engineering Xi'an University of Technology Xi'an 710048 China; ^8^ Division of Nanomaterials and Jiangxi Key Lab of Carbonene Materials Jiangxi Institute of Nanotechnology Nanchang 330200 China; ^9^ Guangdong Institute of Semiconductor Micro‐nano Manufacturing Technology Foshan 528225 China

**Keywords:** Li ion desolvation, lithium metal battery, low temperature surrounding, metal–organic frameworks, sieving effect

## Abstract

Lithium metal anode is desired by high capacity and low potential toward higher energy density than commercial graphite anode. However, the low‐temperature Li metal batteries suffer from dendrite formation and dead Li resulting from uneven Li behaviors of flux with huge desolvation/diffusion barriers, thus leading to short lifespan and safety concern. Herein, differing from electrolyte engineering, a strategy of delocalizing electrons with generating rich active sites to regulate Li^+^ desolvation/diffusion behaviors are demonstrated via decorating polar chemical groups on porous metal–organic frameworks (MOFs). As comprehensively indicated by theoretical simulations, electrochemical analysis, in situ spectroscopies, electron microscope, and time‐of‐flight secondary‐ion mass spectrometry, the sieving kinetics of desolvation is not merely relied on pore size morphology but also significantly affected by the ─NH_2_ polar chemical groups, reducing energy barriers for realizing non‐dendritic and smooth Li metal plating. Consequently, the optimal cells stabilize for long lifespan of 2000 h and higher average Coulombic efficiency, much better than the‐state‐of‐art reports. Under a lower negative/positive ratio of 3.3, the full cells with NH_2_‐MIL‐125 deliver a high capacity‐retention of 97.0% at 0.33 C even under −20 °C, showing the great potential of this kind of polar groups on boosting Li^+^ desolvation kinetics at room‐ and low‐temperatures.

## Graphical abstract

On the basis of pore sieving effect toward Li^+^ desolvation, the intentionally‐introduced polar electron‐donated chemical groups on metal–organic frameworks is developed to further boost Li^+^ desolvation and Li^0^ diffusion kinetics under low temperature, achieving uniform lithium distribution without dendrite formation. As a result, the high capacity‐retention ratio of 97% is stabilized under negative/positive ratio of 3.3 and lower temperature surroundings.

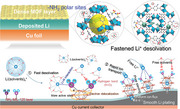

## 1 Introduction

**Figure 1:**
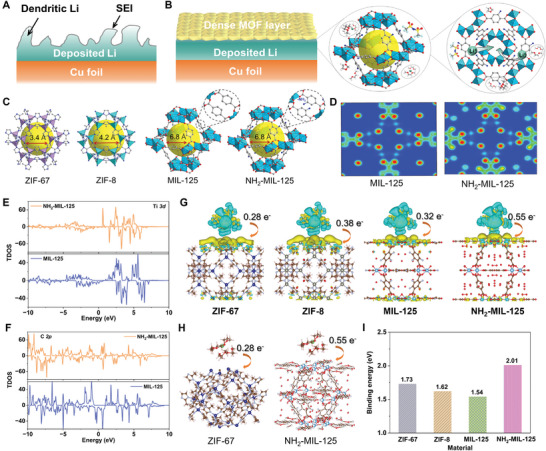
The schematic illustration of plated Li behaviors on A) Cu foil and B) dense MOF layer decorated Cu foil surface. C) Different crystal structures of ZIF‐67, ZIF‐8, MIL‐125, and NH_2_‐MIL‐125. D) The changes of electron density with the introduction of electron‐donor group of NH_2_^−^ in MIL‐125. E,F) The TDOS evolution of Ti 3d and C 2p in MIL‐125 and NH_2_‐MIL‐125. G,H) ZIF‐67, ZIF‐8, MIL‐125 and NH_2_‐MIL‐125 interact with Li(DME)_4_^+^. I) The binding energies of solvated Li(DME)_4_^+^ on different MOFs.

The daily‐increasing demands on sustainable high‐energy‐density lithium‐ion batteries (LIBs) have aroused great interests since rapid developments of electric vehicles and green electric grids.^[^ ^1^ ^]^ Compared to commercial graphite anode in LIBs, metallic Li anode with higher theoretical specific capacity (3860 vs 372 mAh g^−1^) and the lowest electrochemical redox potential (−3.04 V vs SHE) is considered to be the most promising candidate for future Li metal batteries (LMBs).^[^ ^2^ ^]^ However, the Li metal anode also suffers from uncontrollable Li plating behaviors with dendrite formation and the crack of solid electrolyte interphase (SEI).^[^ ^3^ ^]^ Generally, Li deposition morphology basically depends on the kinetics of Li(solvents)*_x_* ^+^ dissociation and Li^+^/Li^0^ transport/diffusion processes from bulk electrolyte to anode surface.^[^ ^4^ ^]^ Reducing the environmental temperature down to low temperature above or around the freezing point, the electrolyte remains liquid and the corresponding solvation shell of Li(solvents)*_x_* ^+^ is inevitably getting larger and larger, and the diffusion kinetics becomes much harder, thus the Li^+^ diffusion in the electrolyte phase is only slightly retarded by the decreased temperature.^[^ ^5^ ^]^ Meanwhile, the uneven spatial distribution of dendrite growth and the formation of dead Li resulted from the sluggish Li^+^/Li^0^ kinetics under low temperatures are used to inducing internal short‐circuits (**Figure** **1**A), shortening cycling lifespan and degrading capacities of LMBs.

In order to address Li dendrite, tremendous efforts of tuning SEI architectures or modulating electrolyte ingredients with different additives in room or low‐temperature conditions have been devoted.^[^ ^6^ ^]^ As known, dendrite growth can kinetically be regarded as the local aggregation of Li atom on the metallic Li surface, where interfacial desolvation for achieving free Li^+^ plays the requisite role before getting electron to form Li atoms under low temperature. Although the popular electrolyte engineering does modulate the solvation shell structure for lower desolvation barriers in the bulk electrolytes, this methodology seems to sacrifice the Li‐ion transport in the low temperature with the presence of co‐solvents.^[^ ^7^ ^]^ Alternatively, metal‐organic frameworks (MOFs) with ordered pore structures and wettability properties provide the chances for sieving large Li(solvents)*_x_* ^+^ clusters and regulating Li^+^ transport, which have attracted increasing attentions.^[^ ^8^ ^]^ However, current designs of reported MOFs in the batteries are random, and there lacks systematic investigation of pore size distribution and chemical groups on the effect of modulating the desolvation kinetics.^[^ ^9^ ^]^ Nobody knows whether it is absolutely correct that smaller pore sizes are benefit for the Li(solvents)*_x_* ^+^ since the Li^+^ is too small (Figure S1, Supporting Information). Meanwhile, in most reported cases, the efficiency of Li^+^‐solvents breaking is strongly dependent on the strength of built‐in physical electric field in the battery, which is the main driving force to propel dissociation.^[^ ^10^ ^]^ How to efficiently accelerate the desolvation kinetics remains to be answered in the low‐temperature surroundings.

Recently, electron delocalization engineering is capable of redistributing electron density, providing more active sites in propelling and dissociating interfacial desolvation for uniformizing free Li^+^ and Li^0^.^[^ ^11^ ^]^ For example, the functionality of the electron‐assisted defect‐rich and/or atomic catalysts have been developed for regulating Li kinetic behaviors.^[^ ^12^ ^]^ Thus, employing electron‐donor polar sites into MOFs is desirable to regulate pore size screening and electronic density of MOFs for highly effective sieving and modulating plating behaviors.^[^ ^13^ ^]^ For the practical application, current studies of metallic Li anodes are based on the presence of excessive Li, which is not suitable for pursuing high energy density.^[^ ^14^ ^]^ Additionally, whether the proposed strategies working efficiently under low temperatures is also uncertain or failed at very low negative/positive (N/P) ratio since the presence of larger‐size solvation sheath structure and slow Li^+^ mobility. Therefore, it is imperative to effectively screen the solvation structure of Li ion at low temperature for Li metal batteries with higher energy density.^[^ ^15^ ^]^

In this work, a detailed combined investigation of pore sieving and electronic density rearrangement of polar chemical groups is pioneered to enhance the desolvation efficiency and interfacial transport kinetics of Li^+^ to promote smooth Li plating at low temperatures. Specifically, a serials of pore size distributions derived from different MOFs and their optimizations with electron‐donor chemical groups are screened and coated on the copper (Figure 1B). As revealed by electrochemical analysis, in situ spectroscopies, electron microscope and time‐of‐flight secondary‐ion mass spectrometry (TOF‐SIMS) as well as theoretical simulations, the effects of synergistic sieving resulting from delocalizing electrons have been comprehensively investigated for generating rapid migration kinetics of Li^+^ and Li^0^ at the interface, realizing non‐dendritic and smooth Li metal plating. Consequently, with limited pre‐plating capacity of 5 mAh cm^−2^ on the MOF‐decorated Cu electrodes (denoted as MOF/Cu@Li), the excellent Li plating/stripping behaviors and extremely lower nucleation overpotentials are achieved, which is attributed to the decreased dissociation barrier of the complex and atomic diffusion to produce free Li^+^. Especially under severe conditions of high mass‐loading or low‐temperature environment, the as‐prepared full cell with NH_2_‐decorated MOFs exhibits superior electrochemical performance with 90.5% capacity retention for 300 cycles under 0 °C and low N/P ratio of 3.3. Even decreasing the temperature down to −20 °C, the capacity‐retention of 97% is maintained after 130 cycles at 0.33 C, paving the way for the practical application of the low‐temperature Li metal battery.

## 2 Results and Discussion

The porous structure of MOF itself, as an effective ionic sieve, can selectively extract Li^+^ and provide uniform Li^+^ flux. To well elucidate the functions of polar group in NH_2_‐MIL‐125, MIL‐125, and other two MOFs (ZIF‐67, ZIF‐8) with smaller pore size distribution were selected here (Figure 1C). Terminal functional groups of polar ─NH_2_ sites may have great influences on electronic properties when absorbing other molecules, and thus changing the kinetics of electron transfer. Initially, the density functional theory (DFT) simulations were used to imitate the changes of electron density with the introduction of electron‐donor groups of ─NH_2_ on MIL‐125 (Figure 1D; Figure S2, Supporting Information). Obviously, the abundant electron density overlaps in NH_2_‐MIL‐125 case in comparison to that of MIL‐125, which will dramatically reduce the desolvation energy barrier. Moreover, the delocalized electron will also bring about the disturbance of neighboring Ti─C bond. As depicted in total density of states (TDOS) of Figure 1E,F, both the Ti *3d* and C *2p* show the separated and broad peaks near the Fermi‐level, implying the presence of electron delocalization for generating more active sites. Owing to the presence of large number of so‐called solvent‐separated ion pair (SSIP) in the moderate electrolyte system, the Li(DME)_4_^+^ is selected to simplify the simulation. As depicted in Figure 1G,H, the charge transfer interaction and capability on grabbing Li^+^ from Li(DME)_4_^+^ was observed when introducing polar ─NH_2_ chemical groups. Figure 1I and Figure S3 (Supporting Information) show the binding energies of solvated Li(DME)_4_^+^ on different MOFs, and the MOFs with smaller size display the higher binding capability, while it works oppositely when the polar sites introduced in the MIL‐125. That is to say, the pore size is not the only index that evaluates the desolvation degree. The NH_2_‐MIL‐125 one exhibits 1.31 times higher than the original one, which is also better than the ZIF systems. Meanwhile, the adsorption behaviors of TFSI^−^ anion on the various MOFs are also investigated in Figure S4 (Supporting Information), and the NH_2_‐MIL‐125 still exhibits the highest adsorption energy of 1.02 eV than others, indicating most TFSI^−^ anions are also adsorbed by the NH_2_‐MIL‐125, accelerating Li ion transference number and ion transport.

**Figure 2:**
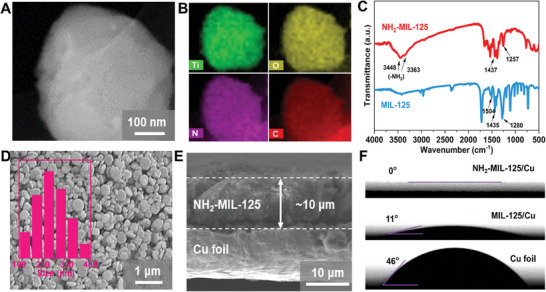
A) TEM image of single NH_2_‐MIL‐125 particle and B) corresponding EDX elemental mappings of Ti, O, N, and C. C) FTIR spectra of NH_2_‐MIL‐125 and MIL‐125. D) Top‐view SEM image of NH_2_‐MIL‐125/Cu. Inset shows the particle size distribution of bending NH_2_‐MIL‐125/Cu. E) The cross‐sectional SEM image of NH_2_‐MIL‐125/Cu with the thickness of 10 µm. F) Contact angles of ether‐based electrolytes on Cu foil, MIL‐125/Cu, and NH_2_‐MIL‐125/Cu electrodes, respectively.

The synthesis of MOFs with polar ─NH_2_ electron modulated groups was displayed in Supporting Information. The transmission electron microscope (TEM) image and corresponding energy dispersive X‐ray spectra (EDX) suggest the sheet‐like structure with uniform distribution of Ti, O, N, and C elements in the as‐obtained NH_2_‐MIL‐125 (**Figure** **2**A,B).^[^ ^16^ ^]^ The X‐ray diffraction (XRD) patterns demonstrate the similar crystal structure of NH_2_‐MIL‐125 and MIL‐125 (Figure S5, Supporting Information); while the characteristic peaks at 3363 and 3448 cm^−1^ in the Fourier transform infrared (FTIR) spectra, assignable to symmetric and asymmetric stretching, suggest the presence of ─NH_2_ polar groups in the NH_2_‐MIL‐125 (Figure 2C).^[^ ^17^ ^]^ After coating on the copper foil, the NH_2_‐MIL‐125 keeps the plate morphology with a size of ≈242 nm (Figure 2D; Figure S6, Supporting Information), which is similar to other MOF‐decorated Cu foils (Figures S7 and S8, Supporting Information). However, it should be noted that the color of these MOF layer is quite different from each other and the coating thickness of MOFs layer is ≈10 µm (Figure 2E). As shown in Figure 2F, the contact angles of Cu foil and MIL‐125 layer are 46° and 11°, respectively. In contrast, the NH_2_‐MIL‐125 layer possesses better affinity and wettability with a contact angle of almost 0°, showcasing the wettability toward electrolyte after considerable polar chemical groups.^[^ ^18^ ^]^

**Figure 3:**
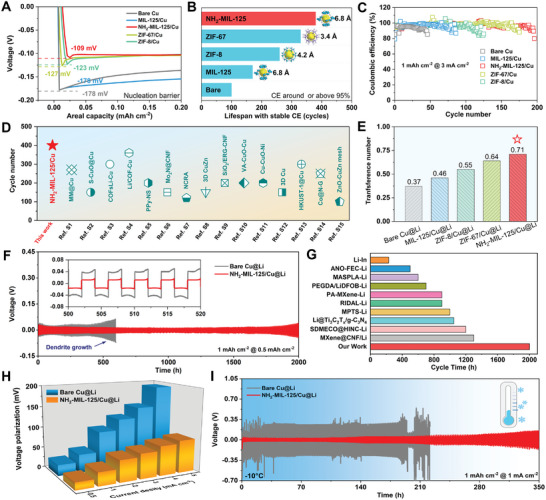
A) Comparison of Li nucleation barriers on the bare Cu, MIL‐125/Cu, NH_2_‐MIL‐125/Cu, ZIF‐67/Cu, and ZIF‐8/Cu electrodes at 1 mA cm^−2^. Comparisons of cycle stability and CEs at B) 1 mA cm^−2^ and C) 3 mA cm^−2^. D) Comparison of cycling stability in half‐cells at 1 mA cm^−2^ for NH_2_‐MIL‐125/Cu with literature reports. E) Comparison of Li ion transference number with different MOFs. F) Cycling performance of bare Cu@Li and NH_2_‐MIL‐125/Cu@Li symmetric cells at 0.5 mA cm^−2^. G) Lifespan comparison between our study and the recently reported literature. H) The comparisons of rate performance of the two symmetric cells at specific current densities. I) Cycling performance of two symmetric cells at 1 mA cm^−2^ under a low‐temperature environment of −10 °C.

To well study the sieving effect of MOF layers coming from pore sizes or polar groups, these MOF‐modified Cu were compared in Li‐Li and Li‐Cu cells. **Figure** **3**A depicts that the nucleation barrier for NH_2_‐MIL‐125 is 109 mV, which is much lower than that of ZIF‐8 (123 mV), ZIF‐67 (127 mV), MIL‐125 (178 mV), and bare Cu (178 mV) systems, suggesting the best desolvation and diffusion kinetics in NH_2_‐MIL‐125 system. Furthermore, the Coulombic efficiencies (CEs) of Li‐Cu cells with/without MOF layers were also compared under 1 or 3 mA cm^−2^ (Figure S9A,B, Supporting Information). As summarized in Figure 3B, the NH_2_‐MIL‐125 system displays the longest lifespan of ≈380 cycles with stable CE (CE >95%), much longer than ZIF‐67 (330 cycles), ZIF‐8 (260 cycles), MIL‐125 (170 cycles), and bare Cu (100 cycles) under 1 mA cm^−2^. Correspondingly, the lowest overpotential in NH_2_‐MIL‐125 system is ≈60.2 mV at 100^th^ cycle (Figure S10, Supporting Information), suggesting the function of this ─NH_2_ polar groups on accelerating the charge transfer kinetics. Increasing the plating/stripping current density to 3 mA cm^−2^, this NH_2_‐MIL‐125 system still behaves the best among all MOF systems and bare Cu, as evidenced by the high CEs after 150 cycles (Figure 3C). Compared with recently impressive results, the NH_2_‐MIL‐125/Cu electrode shows superior performance in lifespan (Figure 3D), demonstrating the high potential of NH_2_‐MIL‐125/Cu electrode for practical LMB systems (Table S1, Supporting Information).

As discussed above, the solvation shell also contains the larger TFSI^−^ anion, that is to say, there exists two different forms of TFSI^−^ anion in the electrolyte, namely, free TFSI^−^ and coordinated TFSI^−^ in the solvation shell of Li^+^, where only the free TFSI‐ contributes to the mobility of total transference (t_anion_+ t _Li+_ = 1). The Li^+^ transference number (*t*_Li+_) can well reflect Li^+^ diffusion kinetics and the *t*_Li+_ of NH_2_‐MIL‐125 system is calculated to be 0.71, much higher than ZIF‐67 (0.64), ZIF‐8 (0.55), MIL‐125 (0.46) and bare Cu system (0.37) (Figure 3E; Figure S11, Supporting Information), verifying the fast desolvation behavior of Li(DME)_4_^+^ on the polar ─NH_2_ decorated MOF. For plating/stripping capability test, the 5 mAh cm^−2^ of Li is electrochemically pre‐plated on the Cu foil or MOFs@Cu. Figure 3F shows bare Cu@Li cell can only survive ≈600 h. In sharp contrast, this NH_2_‐MIL‐125/Cu@Li cell exhibits remarkable cycling stability at 0.5 mA cm^−2^, as evidenced by lasting over 2000 h without any short‐circuit. As highlighted, the polarization gap is decreased from ≈41.2 mV to ≈15.7 mV, only one‐third of the bare Cu@Li case. Increasing the current density to 1 or 5 mA cm^−2^ (Figures S12 and S13, Supporting Information), the NH_2_‐MIL‐125/Cu@Li can stabilize for 1000 or 500 h with lower overpotentials, respectively. Unfortunately, the bare Cu@Li goes up from the beginning cycles and fails suddenly, indicating the serious dendrite formation. To further evaluate the sieving functions of NH_2_‐MIL‐125, a bunch of reported state‐of‐the‐art Li anodes have been summarized in Figure 3G and Table S2 (Supporting Information), the NH_2_‐MIL‐125/Cu@Li anode presents impressive cycling lifespan among various strategies modulated Li metal anodes. The voltage polarizations of symmetric cells under different current densities (0.5–5 mA cm^−2^) are compared in Figure 3H and Figure S14 (Supporting Information). The lower polarization resistances were exhibited in NH_2_‐MIL‐125/Cu@Li especially under high current density, suggesting fast charge and ion transfer across the interphase.

Considering the seriously severe solvation structure under a harsh environment of −10 °C, the symmetric cells were also tested. Figure 3I and Figure S15 (Supporting Information) illustrate bare Cu@Li, ZIF‐67/Cu@Li and MIL‐125/Cu@Li cells behave irregular voltage oscillation due to the sluggish Li^+^ diffusion kinetics, especially the tough desolvation process at interphase under harsh environment. Obviously, the ZIF‐67/Cu@Li system exhibited the barrier of 176 mV, which is lower than that in MIL‐125 system (241mV), indicating the better sieving effect of smaller pore morphology. In contrast, with the superior charge transfer kinetics of sieving between polar ─NH_2_ sites and Li(DME)_4_^+^, NH_2_‐MIL‐125/Cu@Li presents excellent cycling stability over 350 h (Figure 3I). Based on the above results, it can be found the significant influence of NH_2_‐MIL‐125 in accelerating the desolvation of Li^+^ and promoting Li^0^ atom horizontal diffusion toward smooth plating instead of vertical Li dendrite growth. To observe this directly, the morphology evolutions of Li plating under low temperatures were tested by SEM. As shown in Figure S16A (Supporting Information), the formation of Li dendrites is observed as circled in the high‐resolution SEM image on the bare Cu@Li surface. Meanwhile, it can be clearly found that lots of cracks as highlighted on the cycled MIL‐125/Cu@Li surface (Figure S16B, Supporting Information). In contrast, a dense and uniform Li metal surface can be observed in NH_2_‐MIL‐125 system (Figure S16C, Supporting Information), highlighting the effectiveness of the ‐NH_2_ catalytic polar sites in promoting desolvation and atom diffusion. Meanwhile, the interfacial resistance of optimized cell is further reduced along with cycle increase under a low‐temperature environment of −10 °C (Figure S17A, Supporting Information). Due to the introduction of ─NH_2_ polar groups, the Li desolvation and plating behaviors were regulated, and the charge transfer kinetics was modulated at low‐temperature conditions. Also, the Tafel curves in Figure S17B,C (Supporting Information) display the exchange current densities of 0.13, 0.23, 0.24, 0.25, and 0.35 mA cm^−2^ for the five electrodes under a low‐temperature environment of −10 °C, indicating that the NH_2_‐MIL‐125 system has enhanced Li^+^ transfer kinetics.

**Figure 4:**
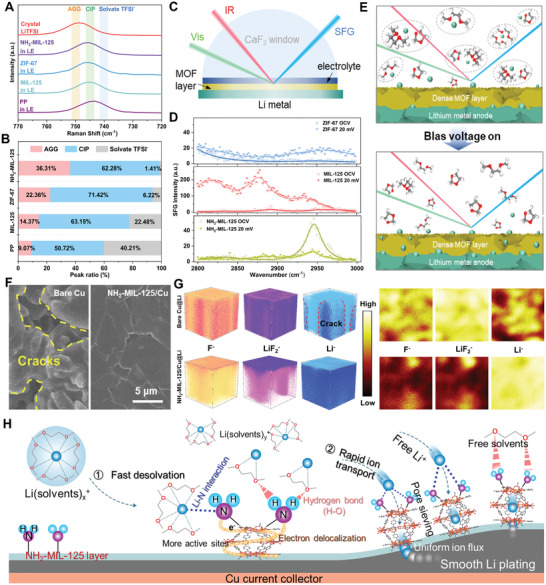
A) Raman spectra of liquid electrolyte on the PP, MIL‐125, ZIF‐67, and NH_2_‐MIL‐125 electrolytes and B) corresponding quantification results of the TFSI^−^ anion states at different interfaces. C) Schematic illustration of the in situ SFG in probing the electrolyte/catalyst interface. D) The SFG evolution of Li^+^ solvation structure under different MOFs with/without bias voltage. E) The illustration of the Li^+^ solvation structure in the electrode/electrolyte interface before and after turning bias voltage on. F) The morphology images of the bare Cu and NH_2_‐MIL‐125/Cu after plating 10 mAh cm^−2^ of Li. G) TOF‐SIMS 3D and 2D spectra of F^−^, LiF_2_^−^, and Li^−^ species of the cycled Cu@Li and NH_2_‐MIL‐125/Cu@Li. H) The schematic illustration of the “capture‐then‐sieving” mechanism of NH_2_‐MOFs on promoting the dissociation of Li(solvents)*_x_* ^+^.

With the assistance of Raman, in situ interface‐sensitive sum frequency generation (SFG) spectroscopy, FTIR and nuclear magnetic resonance (NMR), the desolvation process of Li(DME)_4_^+^ on the modified MOFs were well investigated. Indeed, the MOF layer with abundant pores could well sieve coordinated molecules with specific pore size, exhibiting high ratios of contact ion pairs (CIPs) and aggregated ion pairs (AGGs) (**Figure** **4**A,B; Figure S18, Supporting Information). In sharp contrast, the ratio of AGG and CIPs in NH_2_‐MIL‐125 system is much higher than that in ZIF‐67 or pristine electrolyte. In addition, as displayed in Figure S19 (Supporting Information), the higher intensity of Li^+^‐DME is exhibited in the liquid electrolyte while it shows strong intensity of free DME in the NH_2_‐MIL‐125 system. This can be interpreted by the synergetic effect of pore size sieving and the polar groups, accelerating the dissociation of Li^+^‐solvent clusters. Further, to directly witness the desolvation behaviors under the help of polar chemical groups, the in situ SFG spectroscopy was adopted. The schematic illustration of in situ SFG is depicted in Figure 4C, and the characteristic peaks located at 2876 and 2944 cm^−1^ are attributed to the solvents such as free DME and coordinated DME in solvation shell, which is observed in all systems. With the application of bias potential and electric field, it can be expected that more coordinated DME should be adsorbed on the interface due to the electrically‐moving toward to interface (Figure 4D). That means the coordinated Li^+^‐DME species are more sensitive to the electric field than the free DME molecular. Under this circumstance, the SFG peak intensity of DME should be stronger rather than weaker, as shown in our cases of ZIF‐67 and MIL‐125 systems. However, the NH_2_‐MIL‐125 system case does the opposite behavior, as evidenced by its peak density decreases from OCV (45) to 20 mV (≈10). The main reason for this reduced solvent signal in NH_2_‐MIL‐125 system is caused by efficient active‐acting desolvation process contributed by ─NH_2_ polar groups. The sharp contrast between NH_2_‐MIL‐125 system and MIL‐125 system clearly reveals the big difference of active‐acting desolvation mainly affected by polar sites and passive‐acting desolvation modes affected by electric field. The passive‐acting desolvation needs higher energy, while the active‐acting desolvation mode can effectively complete solvation‐depravation even under a lower electric field, such as 20 mV. As illustrated in Figure 4E, with the sieving effect, the dissociation of Li^+^‐solvents takes place quickly on the polar MOF surface, generating more uniform Li^+^ flux across the MOF layer for lateral diffusion and plating. As shown in Figure S20A (Supporting Information), the NH_2_‐MIL‐125 in DME/DOL in FT‐IR spectra show typical DME/DOL solvent vibration, which is very different from LiTFSI in DME/DOL. These results suggest weak interaction between NH_2_‐MIL‐125 and solvents; while strong interaction existing between Li^+^ and solvents. Moreover, when introducing NH_2_‐MIL‐125 and ZIF‐67 into LiTFSI‐DME/DOL electrolyte system, the absorption peaks of NH_2_‐MIL‐125 system take some changes as indicated by purple band in Figure S20B (Supporting Information). These FT‐IR results demonstrate that ─NH_2_ group has some weak influence on solvation behavior in the electrolyte. To further investigate the effect coming from hydrogen bond or Li⋅⋅⋅N interaction, NMR depicts a very slight shifts of the solvents exhibited in ^1^H NMR (Figure S20C, Supporting Information), displays a very weak hydrogen bonding interaction. While, in comparison with the bare electrolyte, the low‐field shifted resonance (higher absolute value) of NH_2_‐MIL‐125/electrolyte suggests that the shielding effect of TFSI^−^ to Li^+^ is reduced due to the coordination of Li^+^ with the ─NH_2_ group in the ^7^Li NMR (Figure S20D, Supporting Information). Since both MIL‐125 and NH_2_‐MIL‐125 contain carboxylated groups, the high‐field shifted peak of MIL‐125/electrolyte indicates that the carboxylated groups are not involved in the Li^+^ coordination, which well reveals the interactions between Li^+^ and NH_2_ polar groups.

The cycled morphology was subjected to ex situ SEM. As the increase of plating capacity from 2 to 10 mAh cm^−2^, severe Li dendrites and cracks were formed on bare Cu surface (Figure 4F; Figure S21A–C, Supporting Information). In contrast, no dendrites can be found in NH_2_‐MIL‐125/Cu surface (Figure S21D,E, Supporting Information) even after platting 10 mAh cm^−2^ (Figure 4F; Figure S21F, Supporting Information); well suggesting the polar group of ─NH_2_ rapidly fastens the desolvation kinetics to achieve the uniform Li deposition behavior. Furthermore, TOF‐SIMS was performed to investigate the 3D structure and surface species. The strong signals of F^−^ and LiF_2_^−^ ion species can be detected in bare Cu system, which is caused by the decomposition of excessive electrolyte. The prolonging sputter time up to 500 s also indicates the thickness of the generated SEI layer is pretty thick, which causes larger polarization resistance. In the 3D reconstruction (Figure 4G; Figure S22A, Supporting Information), although LiF component seems quite abundant, the obvious crack of Li deposition is found without any well distribution. However, for the system of NH_2_‐MIL‐125/Cu@Li, the decomposition fragment signals are decreased after 10 s, suggesting a thin SEI layer; which is also confirmed by 3D and 2D mappings, as evidenced by the thin layer of LiF on top surface with strong intensity and uniform deposition of Li on the bottom (Figure 4G; Figure S22B, Supporting Information). At the same time, XPS analysis was performed after the initial deposition or 50 cycles of the NH_2_‐MIL‐125/Cu@Li electrode. Owing to the pre‐deposition of 5 mAh cm^−2^ (with very limited Li capacity) on substrates such as Cu or MOF/Cu in advance, all Li anodes before cycling already have established the SEI via electrochemical method, which is very different from commercial fresh Li plate. The presence of NH_2_ groups was confirmed by the N 1s and C 1s spectra in the XPS test results (Figure S23A, Supporting Information), and the results were almost consistent with those before cycling (Figure S23B, Supporting Information), which means the chemical groups of ─NH_2_ are stable during plating/stripping process. With the modulation of NH_2_‐MIL‐125 on the Cu without any pre‐plating Li (Figure S23C, Supporting Information), more inorganic lithium‐salt components such as LiF and Li_3_N are formed than that in the other systems, which can be explained by the TFSI^−^ trapping and concentrating effect of the modulation layer.

From above electrochemical and spectroscopical analysis, the MOF layer with functional group of ─NH_2_ is designed to fasten Li^+^ desolvation process via the properly regulated interaction between ─NH_2_ and Li(solvents)*_x_* ^+^, and the desolvation mechanism can be interpreted in Figure 4H and Figure S24 (Supporting Information): 1) The electron‐donor nature of ‐NH_2_ provides the lone‐pair electrons to capture the solvated Li^+^; 2) Consistent with the simulations, the ‐NH_2_ polar sites participate in the electronic density redistribution over the whole MOF skeleton by forming *p‐*–*π* conjugation between the ─NH_2_ group and the benzene ring, realizing electron delocalization and generating more possible sites for Li^+^ to reside temporally and diffuse to the nearby sites smoothly; 3) The formation of hydrogen bond between the ─NH_2_ group and the solvent molecule may play a synergic role for the dissociation of Li(solvents)*_x_* ^+^; 4) Both the chemical adsorbing interaction and physical pore sieving interaction of NH_2_‐MIL‐125 help to dissociate the Li(solvents)*_x_* ^+^ complex rapidly in the system; 5) The generated isolated Li^+^ flux is also sieved by the pores of MOFs to uniformize the local distribution for smooth plating. Above all, the smooth plating behaviors should be attributed to the fast desolvation kinetics enabled by the interactions between ─NH_2_ polar sites and Li(solvents)*_x_* ^+^ together with uniform ion flux.

**Figure 5:**
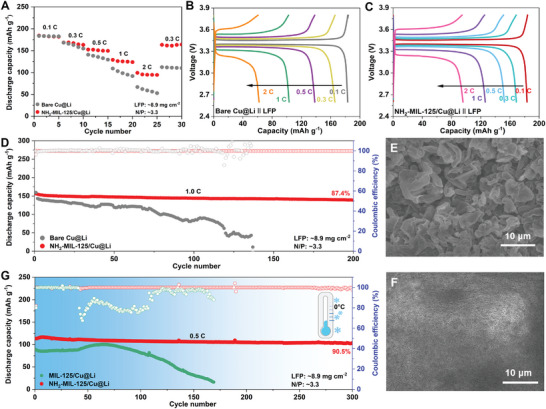
Comparisons of A) rate performance and B,C) corresponding voltage profiles of the two full cells with bare Cu@Li and NH_2_‐MIL‐125/Cu@Li electrodes under N/P ratio of ≈3.3. D) Cycling performance of full cells at 1 C (1 C = 170 mA g^−1^). High‐resolution SEM images of E) bare Cu@Li and F) NH_2_‐MIL‐125/Cu@Li in the cycled full battery. G) Cyclic performance of the full cells with MIL‐125/Cu@Li and NH_2_‐MIL‐125/Cu@Li electrodes at 0.5 C under a low‐temperature environment of 0 °C.

The superior transport kinetics propelled by NH_2_‐MIL‐125/Cu@Li are also evaluated in full cells employed with NH_2_‐MIL‐125/Cu@Li with 5 mAh cm^−2^ as the anode. The Li‐LFP full cell based on NH_2_‐MIL‐125/Cu@Li exhibits a lower charge‐transfer resistance (18.9 Ω) compared to that of bare Cu@Li (56.2 Ω) (Figure S25, Supporting Information), and the corresponding full cell delivers robust rate capability with a low negative/positive (N/P) ratio of 3.3 (**Figure** **5**A). The related voltage/capacity profiles in Figure 5B,C well demonstrate the fast Li^+^ diffusion kinetics in NH_2_‐MIL‐125/Cu@Li system, as revealed by the high capacity‐retention of 68.9% and 52.2% at 1 and 2 C (in relation to the capacity of 183 mAh g^−1^ at 0.1 C), respectively, which are much higher than one in bare Cu@Li system (56.3% and 33.9%). For the long‐term cycling in Figure 5D, the cell based on NH_2_‐MIL‐125/Cu@Li behaves outstandingly stable in both capacity and CE under 1 C and it stabilized 139 mAh g^−1^ with high CE of 99.8% after 200 cycles, corresponding to the capacity retention of 87.4%, better than state‐of‐the‐art electrode reports (Figure S26, Supporting Information). However, one can observe that the full cell based on bare Cu@Li decreased to 49 mAh g^−1^ along with the fluctuated CEs after 120 cycles. Further, in comparison to the significant fragments and a considerable amount of randomly oriented Li dendrites on bare Cu@Li surface (Figure 5E), a dense and uniform Li metal surface can be observed in NH_2_‐MIL‐125 system, highlighting the effectiveness of the NH_2_‐MIL‐125 in promoting desolvation and atom diffusion (Figure 5F; Figure S27, Supporting Information). As known, the electrochemical behaviors of Li‐LFP cells under low‐temperature surroundings are still challenging at high charging/discharging rate. The cell based on NH_2_‐MIL‐125/Cu@Li was assessed under 0 °C. As shown in Figure 5G, this cell with a low N/P ratio of 3.3 can stabilize up to 300 cycles with a high CE of 98.7% at 0.5 C, and it could keep the capacity retention of 90.5% after 300 cycles, much better than MIL‐125/Cu@Li, well suggesting the great potential of NH_2_‐MIL‐125/Cu@Li anode for practical low‐temperature application. Afterward, under 0 °C, in comparison to the severe Li dendrites and cracks on the MIL‐125/Cu@Li surface (Figure S28A, Supporting Information) in the full cell, a smooth and dense Li‐metal surface highlights the effectiveness of the ─NH_2_ in promoting desolvation and atom diffusion (Figure S28B, Supporting Information) in the full cell. These results demonstrate the effect of NH_2_‐MIL‐125 in inhibiting lithium dendrites and dead lithium at both room temperature and low temperature. An overview of the state‐of‐the‐art LiFePO_4_‐based LMBs highlights the superiority of NH_2_‐MIL‐125/Cu@Li electrode in the low temperature environment, which is considerably competitive with these previously reported electrolyte engineering (Figure S29, Supporting Information). Under low temperatures of −20 °C, with the fastened sieving effect of NH_2_‐MIL‐125, both Li‐NCM811 full cells exhibit high performance and electrochemical stability at 0.1, 0.2, and 0.33 C, respectively (Figure S30, Supporting Information). As exhibited, the full cell based on NH_2_‐MIL‐125/Cu@Li exhibited the initial capacity of 159 mAh g^−1^ at 0.1 C. Even enhancing the current rate to 0.2 and 0.33 C, the full cell with NH_2_‐MIL‐125/Cu@Li remained the capacity retention of 98.0% or 97.0% after 90 or 130 cycles, respectively, which is much superior to the bare Cu@Li ones. Compared with recent reports of low‐temperature batteries in Table S3 (Supporting Information), we are delighted to find our results are among the top ones and better than most ones when considering the less N/P ratio.

## 3 Conclusion

In summary, with the synergistically sieving effect of polar ─NH_2_ groups on MOF modified Cu current collector, a strategy of delocalizing electrons with generating more active sites to regulate Li behaviors of Li^+^ desolvation and Li atom diffusion process has been achieved. Comprehensive simulations and in situ/ex situ characterizations reveal the diffusion and desolvation kinetics is not merely relied on pore size morphology but also affected by the polar sites on MOFs. Specifically, the critical roles of ─NH_2_ polar groups in expelling solvent molecules from Li^+^ to reduce the desolvation energy barrier and in injecting electrons to anions for interphase formation under room and low‐temperature conditions are revealed. Consequently, the optimized cells can last for 2000 h and high CEs without any dendrite formation, which is four‐fold extended lifespan of bare Cu system. Impressively, under the lower N/P ratio of 3.3, the full cell with NH_2_‐MIL‐125 displays a high capacity‐retention of 90.5% for 300 cycles under 0 °C. Even under −20 °C, the as‐fabricated Li‐NCM811 can stabilize for 130 cycles with the capacity‐retention of 97% at 0.33 C, indicating its great potential in future application. This facile strategy can be extended to other metal battery systems by tailoring the molecular structures to boost the interfacial diffusion kinetics.

## Conflict of Interest

The authors declare no conflict of interest.

## Supporting information



Supporting Information

## Data Availability

The data that support the findings of this study are available from the corresponding author upon reasonable request.
