# Supplementary material for: Low‐Temperature Lithium Metal Batteries Achieved by Synergistically Enhanced Screening Li+ Desolvation Kinetics
Source: Adv Mater. 2024 Dec 16;37(5):2411601. doi: 10.1002/adma.202411601 (PMC11795707; doi:10.1002/adma.202411601)
Supplement: Supplementary file 1 — Supporting Information [file ADMA-37-2411601-s001.docx]

Supporting Information

Low-Temperature Lithium Metal Batteries Achieved by Synergistically Enhanced Screening Li^+^ Desolvation Kinetics

*Fengyi Zhu^#^, Jian Wang^#,^*, Yongzheng Zhang, Haifeng Tu, Xueqing Xia, Jing Zhang, Haiyan He, Hongzhen Lin, Meinan Liu**

F. Zhu, Prof. M. Liu

State Key Laboratory of Featured Metal Materials and Life-cycle Safety for Composite Structures, Guangxi Key Laboratory of Processing for Non-Ferrous Metals and Featured Materials, School of Resources, Environment and Materials, Guangxi University, Nanning 530004, China

E-mail: meinanliu@gxu.edu.cn

F. Zhu, J. Wang, H. Tu, Prof. H. Lin, Prof. M. Liu

*i*-lab, & CAS Key Laboratory of Nanophotonic Materials and Devices, Suzhou Institute of Nano-Tech and Nano-Bionics, Chinese Academy of Sciences, Suzhou 215123, Jiangsu, China

E-mail: wangjian2014@sinano.ac.cn; mnliu2013@sinano.ac.cn

Dr. J. Wang

Helmholtz Institute Ulm (HIU), Ulm 89081, Germany

Karlsruhe Institute of Technology (KIT), Karlsruhe D-76021, Germany

E-mail: jian.wang@kit.edu

Dr. Y. Zhang

State Key Laboratory of Chemical Engineering, East China University of Science and Technology, Shanghai 200237, China

Dr. J. Zhang

School of Materials Science and Engineering, Xi’an University of Technology, Xi’an 710048, China

F. Zhu, X. Xia, Prof. H. He

College of Mechanics and Materials, Hohai University, Nanjing 210098, China

Prof. M. Liu

Division of Nanomaterials and Jiangxi Key Lab of Carbonene Materials, Jiangxi Institute of Nanotechnology, Nanchang 330200, China.

Prof. M. Liu

Guangdong Institute of Semiconductor Micro-nano Manufacturing Technology, Foshan 528225, China.

**Experimental Section**

***Material preparation***

All chemicals from suppliers were used without any further purification. Titanium isopropoxide (TTIP), terephthalic acid (H_2_BDC), 2-aminoterephthalate (H_2_ATA), Co(NO_3_)_2_·6H_2_O, Zn(NO_3_)_2_·6H_2_O, 2-Methylimidazole (2-MeIM), dimethylformamide (DMF) and ethanol were purchased from Aladdin.

***Material preparation of MIL-125***

3.24 g H_2_BDC was added to 56 mL methanol solution with continuously stirring until the solution was transparent. Afterwards, 1.6 mL TTIP was slowly dropped into the mixture and then transferred to the autoclave and kept at 150 °C for 24 h. The product was collected by centrifuge and washed with deionized water, dimethylformamid (DMF), and ethanol for several times. Lastly, the powder was activated under a heat treatment at 150 °C overnight in a vacuum oven.

***Material preparation of NH_2_-MIL-125***

H_2_ATA (0.816 g) was added to the mixture of 11.25 mL of DMF and 3.75 mL of methanol, then 0.45 mL TTIP was slowly dropped into the mixture and then transferred to a 50 mL autoclave and kept at 150 °C for 24 h. The product was collected by centrifuge and washed with deionized water, DMF, and ethanol several times. Then, the powder was activated by heating overnight at 150 °C in a vacuum oven.

***Material preparation of ZIF-67***

0.55 g Co(NO_3_)_2_·6H_2_O was added to 60 mL methanol solution with continuously stirring until the solution was transparent. Afterward, 4.11 g 2-MeIM was slowly dropped into the mixture and then stirring continuously for 24 h. The product was collected by centrifuge and washed with deionized water, DMF, and ethanol for several times. Then, the powder was activated under a heat treatment at 150 °C overnight in a vacuum oven.

***Material preparation of ZIF-8***

0.625 g Zn(NO_3_)_2_·6H_2_O was added to 30 mL methanol, then 3.55 g 2-MeIM was slowly dropped into the mixture and then transferred to a 50 mL autoclave and kept at 120 °C for 36 h. The product was collected by centrifuge and washed with deionized water, DMF, and ethanol for several times. Then, the powder was activated by heating overnight at 150 °C in a vacuum oven.

***Fabrication of lithium metal cells***

The as-obtained ZIF-8, ZIF-67, MIL-125 and NH_2_-MIL-125 and polyvinylidene fluoride (PVDF) binder were mixed in N-methyl-2-pyrrolidone (NMP) with a mass ratio of 9: 1 to form slurries, respectively, which were then uniformly coated onto the Cu foil via a doctor blade method, followed by desiccation in a vacuum oven at 60 °C for overnight. Afterwards, the Cu foils coated with ZIF-8, ZIF-67, MIL-125 and NH_2_-MIL-125 were punched into disks of 15 mm in diameter, and denoted as ZIF-8/Cu, ZIF-67/Cu, MIL-125/Cu and NH_2_-MIL-125/Cu, respectively. Additionally, bare Cu foil (Bare Cu) was also punch into disks and used for reference. To evaluate the Coulombic efficiency, the as-prepared bare Cu, ZIF-8/Cu, ZIF-67/Cu, MIL-125/Cu and NH_2_-MIL-125/Cu were used as the working electrodes to assemble Li-Cu half cells with Li foils serving as the counter electrode. Symmetric cells were assembled using bare Cu and NH_2_-MIL-125/Cu with pre-plated 5 mAh cm^-2^ Li (denoted as Bare Cu@Li, NH_2_-MIL-125/Cu@Li) to measure the long-term electrochemical behavior of Li plating/stripping. For the half and symmetric cells, the electrolyte was 1 M lithium bis (trifluoromethyl-sulfonyl) imide (LiTFSI) dissolved into a mixture of 1,3-dioxolan (DOL) and 1,2-dimethoxyethan (DME) (1: 1 by volume) containing 2wt% Lithium nitrate (LiNO_3_). As for full-cell tests, commercial LiFePO_4_ (LFP)/LiNi_0.8_Co_0.1_Mn_0.1_O_2_ (NCM811) was chosen as the cathode active material. The LFP powder was mixed with carbon black and PVDF (mass ratio of 8:1:1) in NMP, and then casted onto the carbon-coated Al foil via a doctor blade method with an active-material loading about 8.9 mg cm^-2^, followed by drying under vacuum at 60 °C overnight. The commercial low-temperature electrolyte is an ester electrolyte system with code of LB-141 (from Suzhou Duoduo reagent company), where the 1 M LiPF_6_ is dissolved in carbonate ester and carboxylate ester mixed solutions as well as some special electrolyte additives.

***Li-ion transference number measurement***

The Li ion transference number was determined with chronoamperometry at a constant step potential of 10 mV and the Li-ion transference number was calculated based on the following equation:

$$t_{{Li}^{+}}= \frac{I_{s}(\Delta V-{I_{0}R}_{0})}{I_{0}(\Delta V-{I_{s}R}_{s})}$$

where *t*_Li+_ is transference number; *I*_0_ and *I*_s_ are the initial and steady state current; *R*_0_ and *R*_s_ are the initial and steady state resistances of the passivating layers; Δ*V* is a constant applied polarize potential of 10 mV.

***Material Measurements***

The morphology of sample and cycled electrodes were characterized by using field emission scanning electron microscopy (FE-SEM, Hitachi S4800) or a F20 S-Twin field-emission transmission electron microscope (TEM, Tecnai G2). The MIL-125 and NH_2_-MIL-125 sample was subjected to X-ray diffraction (XRD, Rigaku D/Max 2550) using Cu Kα radiation. The hydrogen bonding effect between NH_2_-MIL-125 and electrolyte were verified by Fourier transform infrared spectroscopy (Nicolet IN 10) and nuclear magnetic resources (NMR, Bruker). Raman spectra (Horiba LabRAM ARAMIS) was used to distinguish anion structures at the electrolyte/anode interface. The SEI layer information after cycling or pre-plated with 5 mAh cm^-2^ was carried out on X-ray photoelectron spectroscopy (XPS, Thermo Scientific Nexsa). The in-situ sum frequency generation (SFG) measurements were performed on the commercial picosecond laser system with/without applying the bias voltage of 20 mV, where the IR pulse ranges from 3000 to 3600 cm^-1^ and visible light wavelength is set at 532 nm. Time-of-flight secondary-ion mass spectrometry (IONTOF) was utilized to measure cycled MOF/Cu@Li surfaces. The voltage curves or cycling performance of Li-Li, Li-Cu and full cells were conducted on a battery test system (LAND CT-2001). Electrochemical impedance spectroscopy (EIS) spectra were obtained on a Bio-logic electrochemical workstation (VMP-3) within the frequency of 100 mHz ~200 kHz.

***Theoretical simulation***

The Vienna Ab initio Simulation Package (VASP) was used to perform DFT calculations within periodic models. A generalized gradient approximation (GGA) with projector augmented wave (PAW) method was applied in calculations to better simulate the electronic structures and the core-electron interaction. The cutoff energy was set to be 450 eV, and the force threshold of optimization convergence was 0.05 eV Å^-1^. In detail, the solvated Li ion cluster consists of a Li ion surrounded by four DME molecules, and the (001) plants of all MOFs are the main surface of adsorption.

**Supplementary Figures**


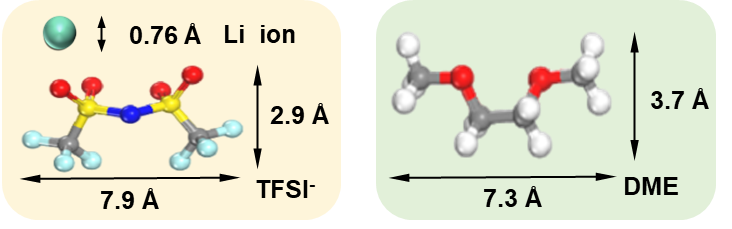


**Figure S1.** The sizes of Li^+^, TFSI^-^ and DME.


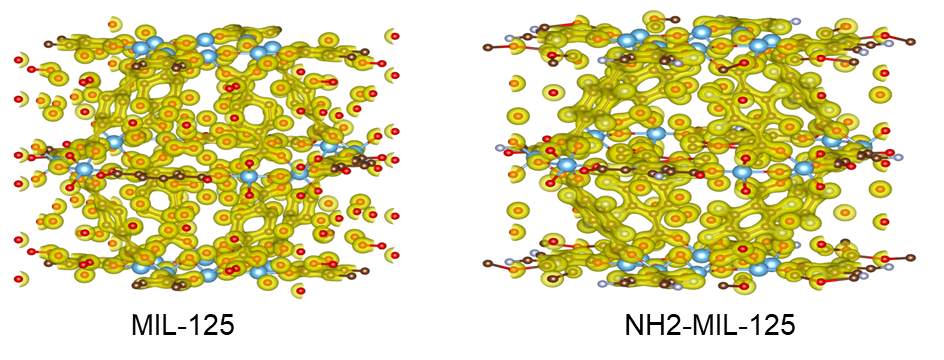


**Figure S2.** The changes of electron density with the introduction of electron-donor group of NH_2_^-^.


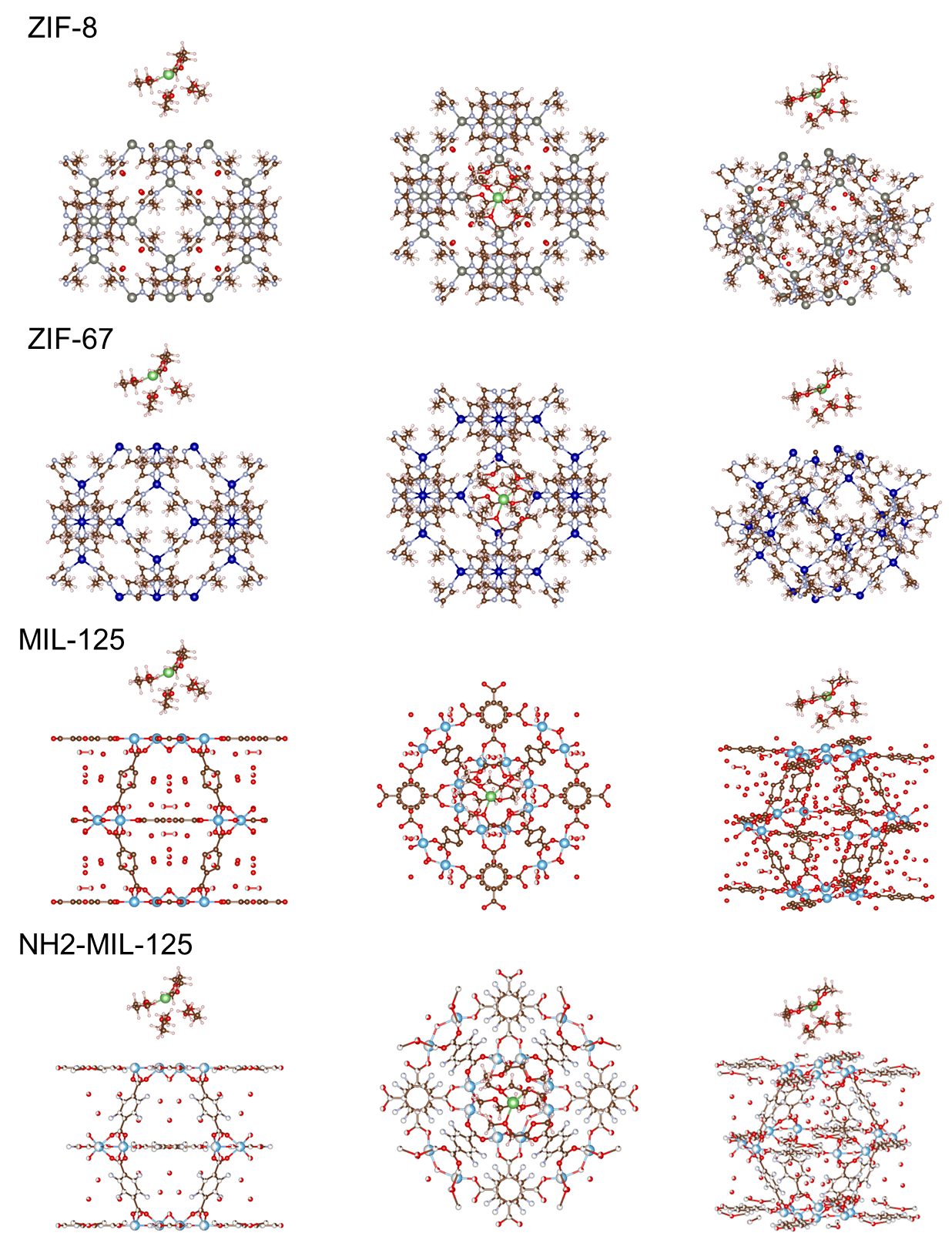


**Figure S3.** The different aspects of solvated Li(DME)_4_^+^ on different MOFs.


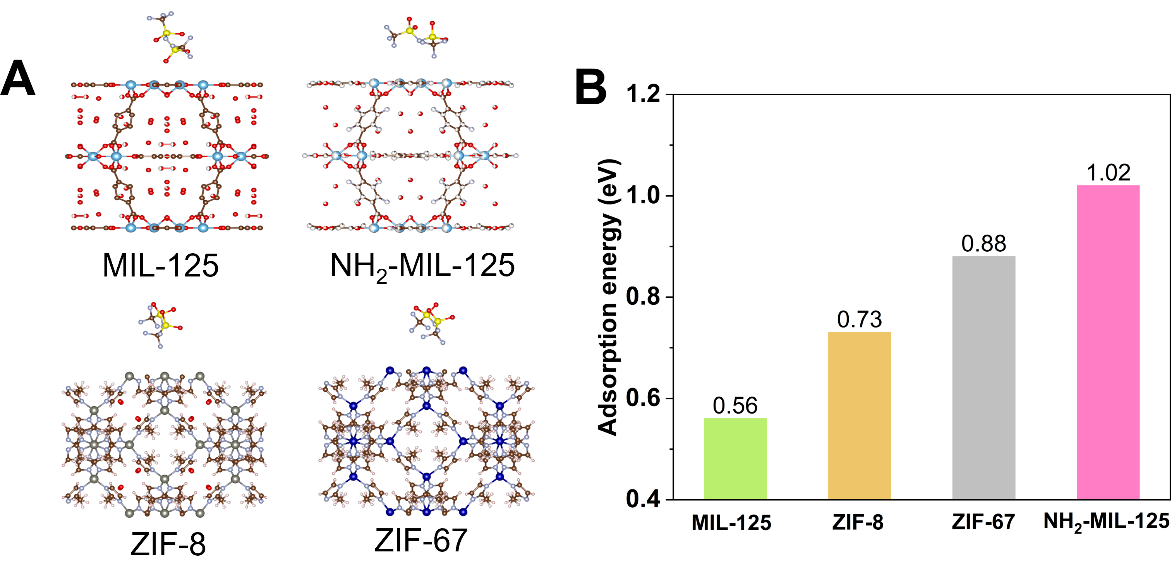


**Figure S4.** (A) The structure and (B) corresponding adsorption energy between different MOFs and TFSI^-^ anion.


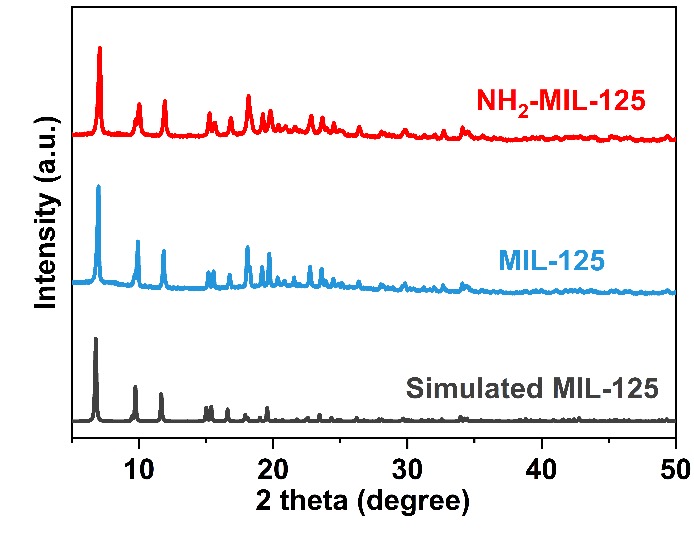


**Figure S5.** The XRD patterns of NH_2_-MIL-125 and MIL-125.


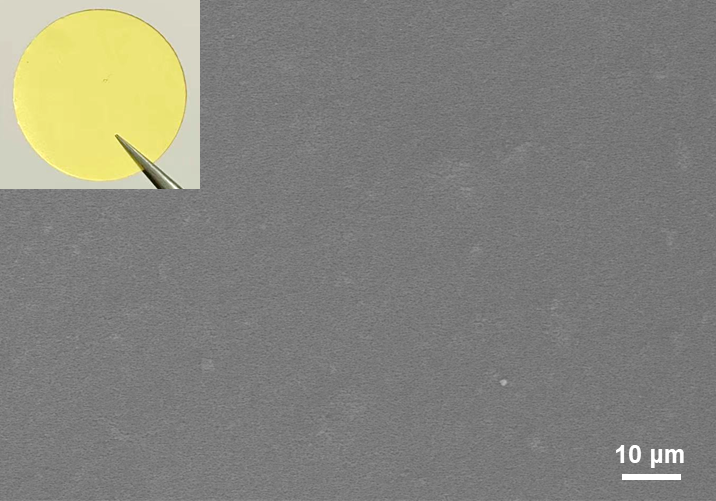


**Figure S6.** Low magnification SEM image of NH_2_-MIL-125 layer on Cu foil.


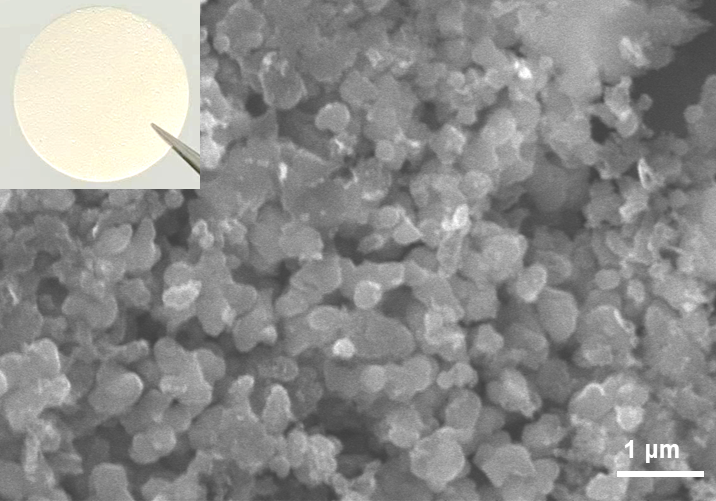


**Figure S7.** High magnification SEM image of MIL-125 on Cu foil.


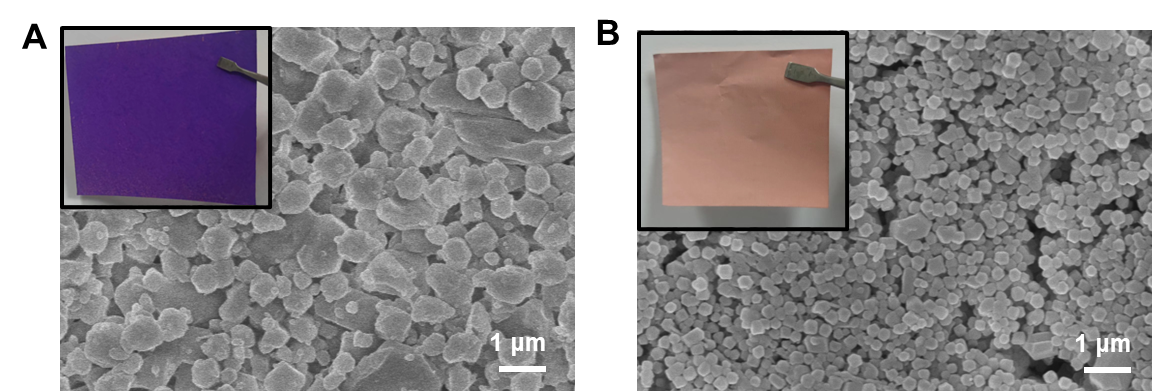


**Figure S8.** High magnification SEM images of (A) ZIF-8 layer and (B) ZIF-67 layer on Cu foil, respectively.


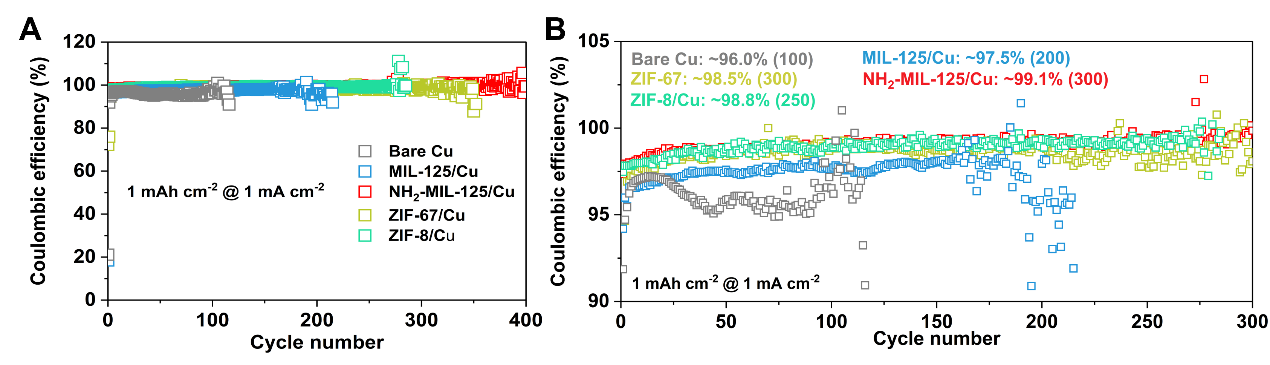


**Figure S9.** Comparison of (A) the CEs and (B) the average CE during the whole cycling for bare Cu, MIL-125/Cu, NH_2_-MIL-125/Cu, ZIF-67/Cu and ZIF-8/Cu in half-cells at 1 mA cm^-2^.


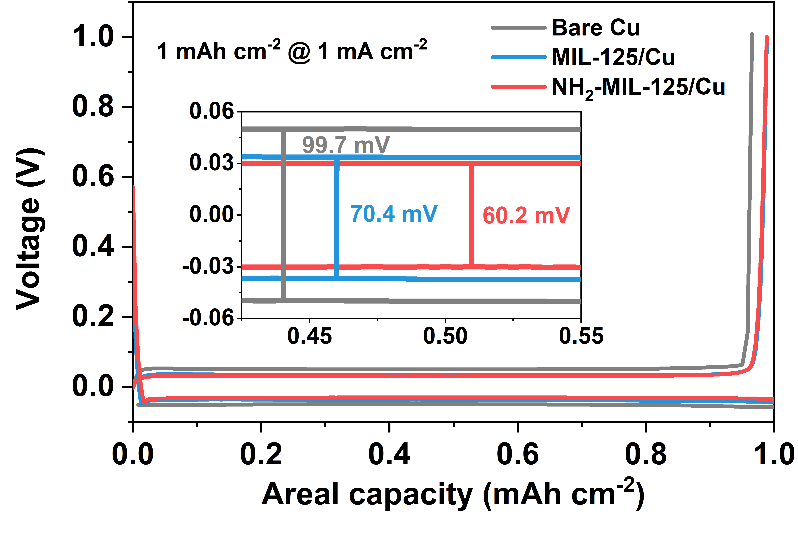


**Figure S10.** Corresponding potential profile comparisons at 100^th^ cycle of the bare Cu, MIL-125/Cu and NH_2_-MIL-125/Cu electrodes at 1 mA cm^−2^.


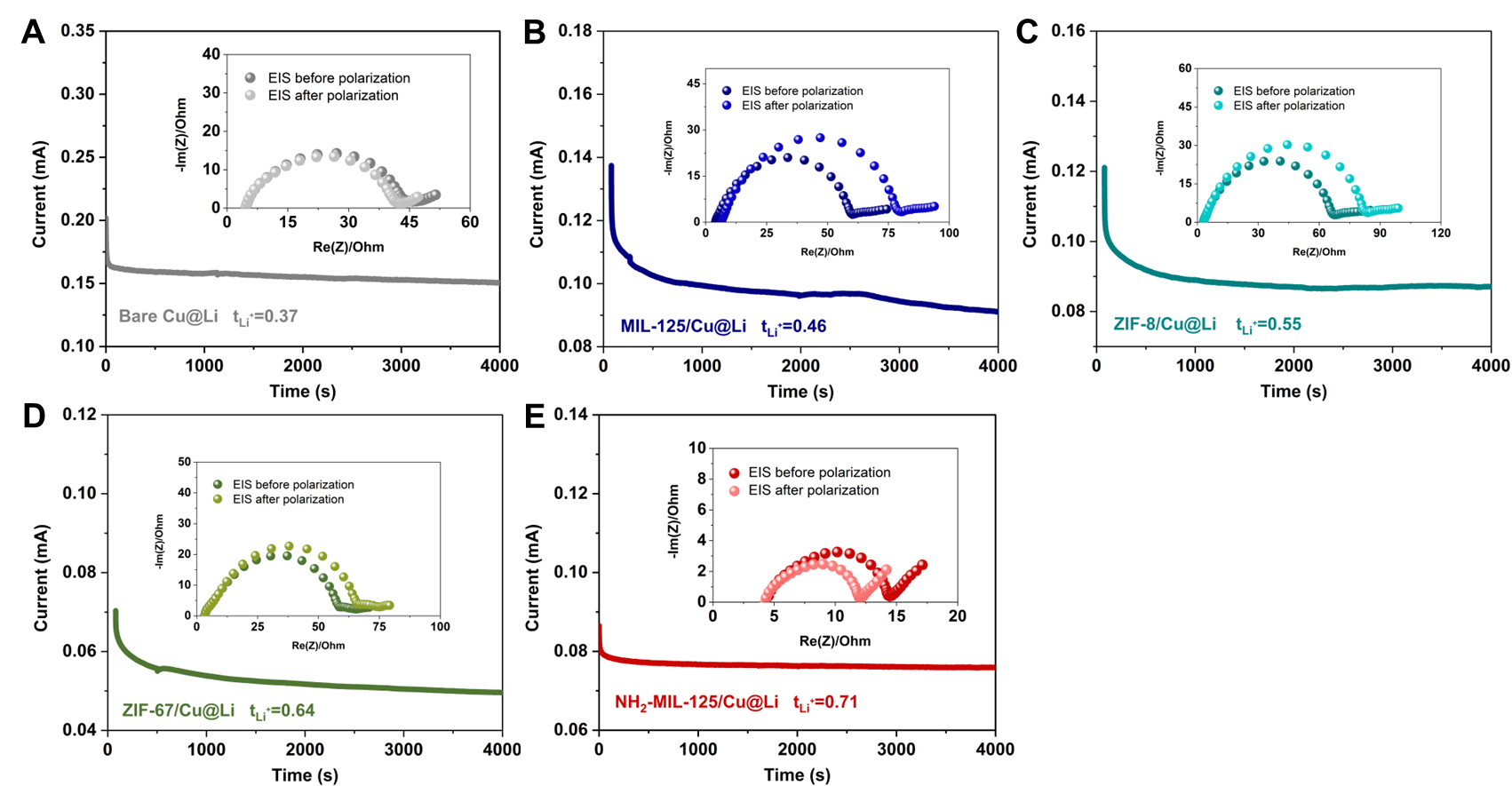


**Figure S11.** Li ion transference number measurement for (A) bare Cu@Li, (B) MIL-125/Cu@Li, (C) ZIF-8/Cu@Li, (D) ZIF-67/Cu@Li and (E) NH_2_-MIL-125/Cu@Li.


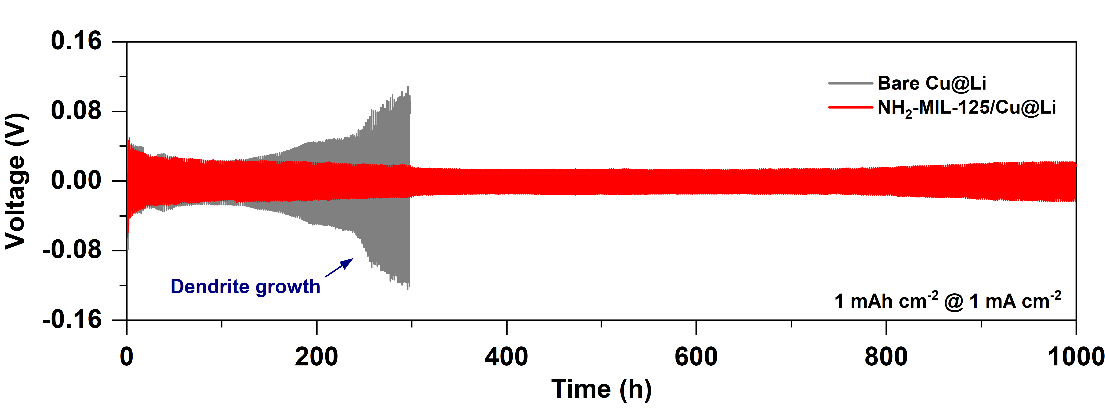


**Figure S12.** Voltage profiles of bare Cu@Li and NH_2_-MIL-125/Cu@Li symmetric cells at 1 mA cm^-2^.


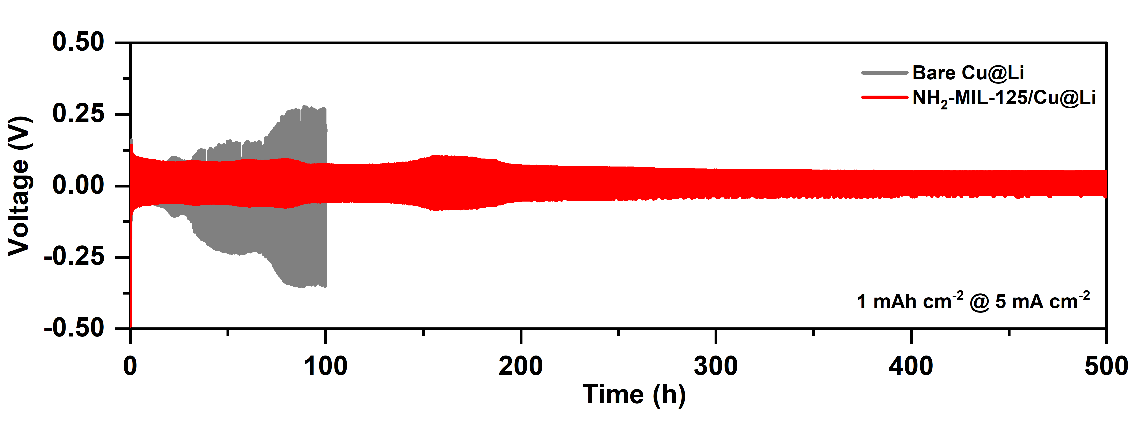


**Figure S13.** Voltage profiles of bare Cu@Li and NH_2_-MIL-125/Cu@Li symmetric cells at a current density of 5 mA cm^-2^.


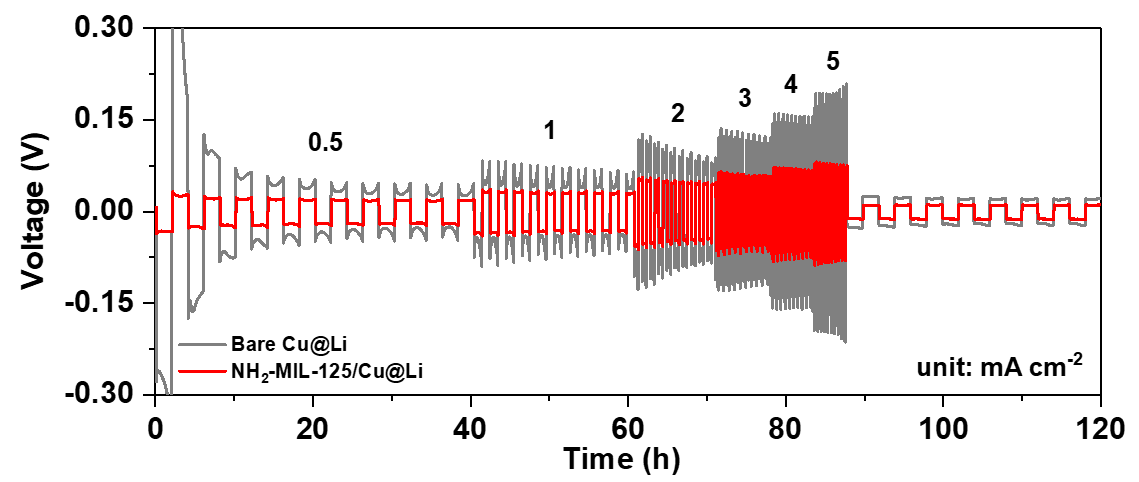


**Figure S14.** The comparisons of rate performance of bare Cu@Li and NH_2_-MIL-125/Cu@Li symmetric cells at specific current densities.


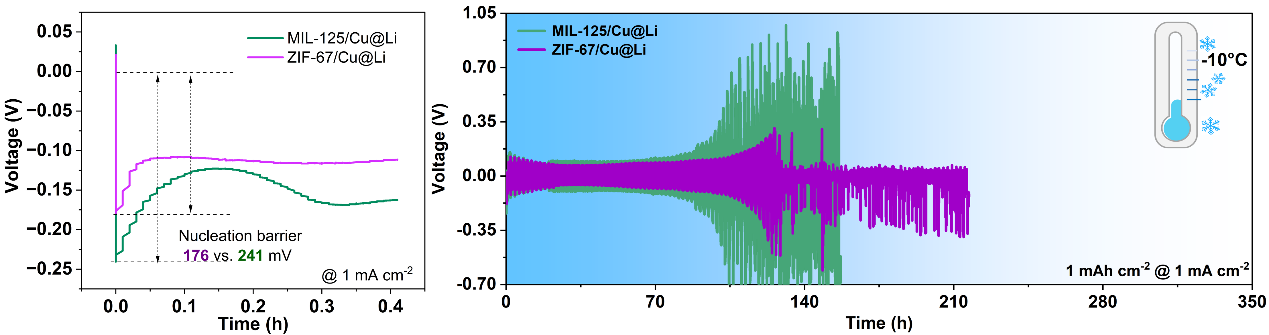


**Figure S15.** Desolvation barriers and Cycling performance of ZIF-67/Cu@Li, MIL-125/Cu@Li and NH_2_-MIL-125/Cu@Li symmetric cells at 1 mA cm^-2^ under a low-temperature environment of -10 °C.


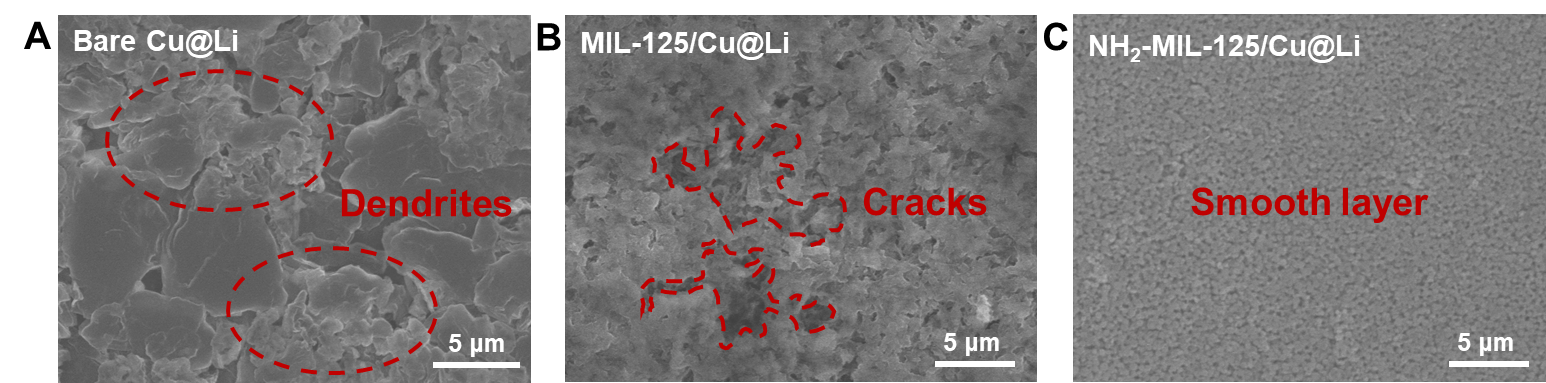


**Figure S16.** High-resolution SEM images of cycled (A) bare Cu@Li, (B) MIL-125/Cu@Li and (C) NH_2_-MIL-125/Cu@Li electrodes under a low-temperature environment of -10 °C.


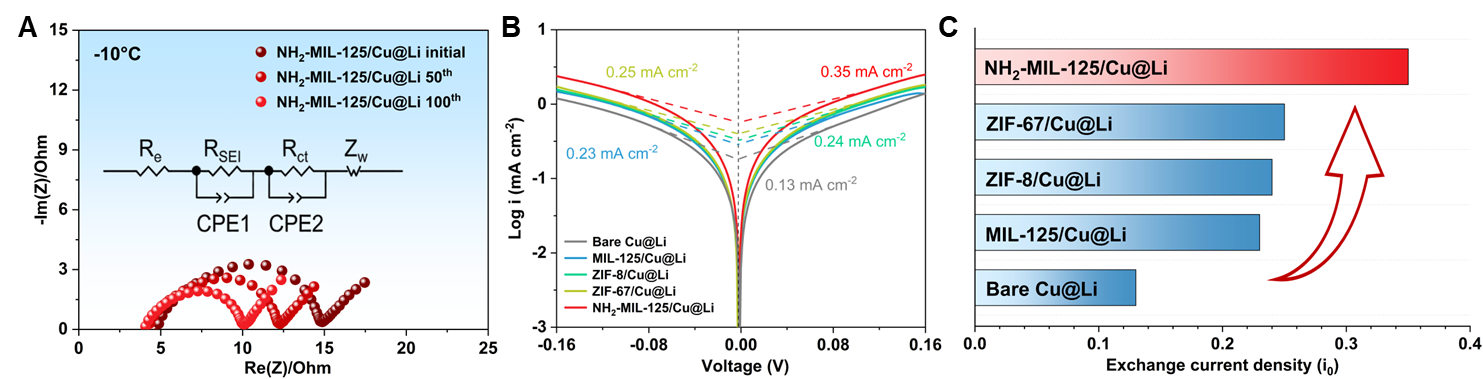


**Figure S17.** (A) Comparison of EIS for NH_2_-MIL-125/Cu@Li electrodes at different cycles under a low-temperature environment of -10 °C. (B) Tafel curves and (C) comparisons of exchange current density for five electrodes under a low-temperature environment of -10 °C.


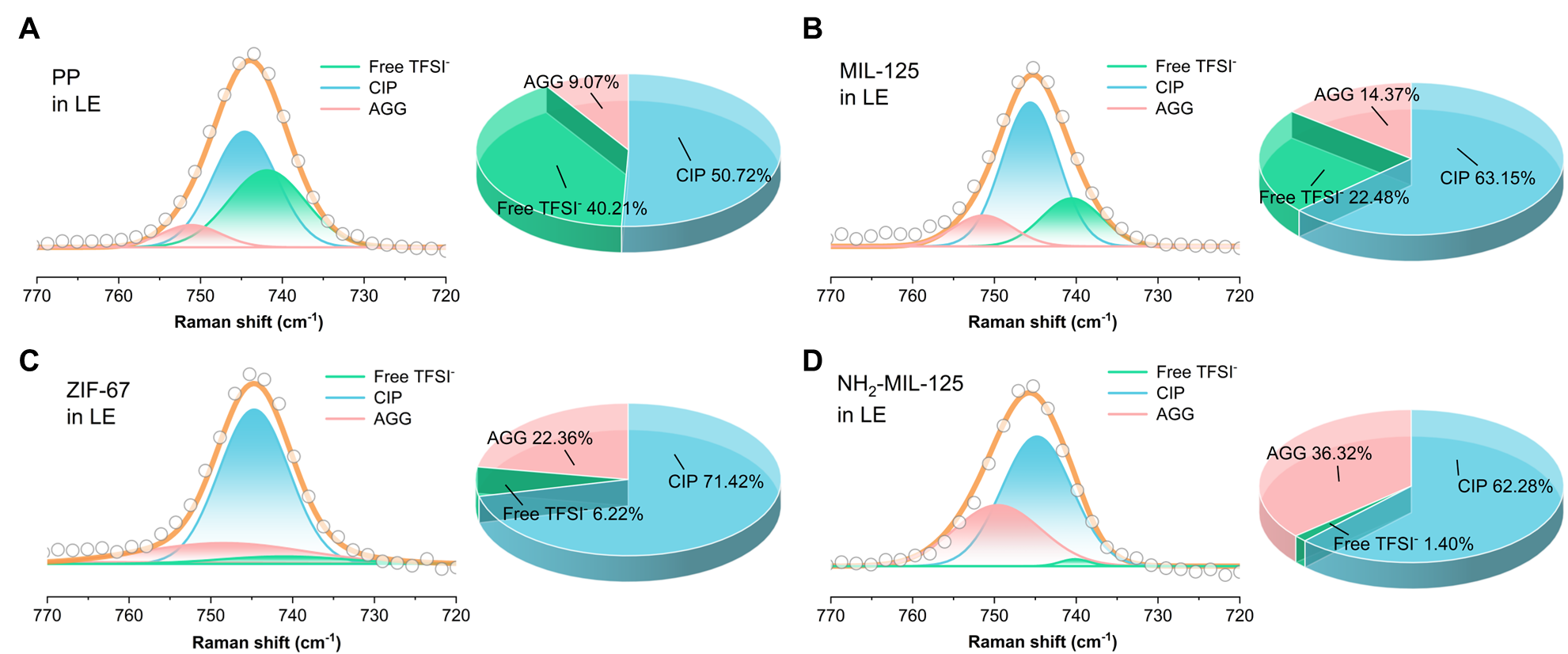


**Figure S18.** Raman spectra of the electrolyte on (A) PP, (B) MIL-125, (C) ZIF-67 and (D) NH_2_-MIL-125 and corresponding quantification results of the TFSI^-^ anion states at the interface.


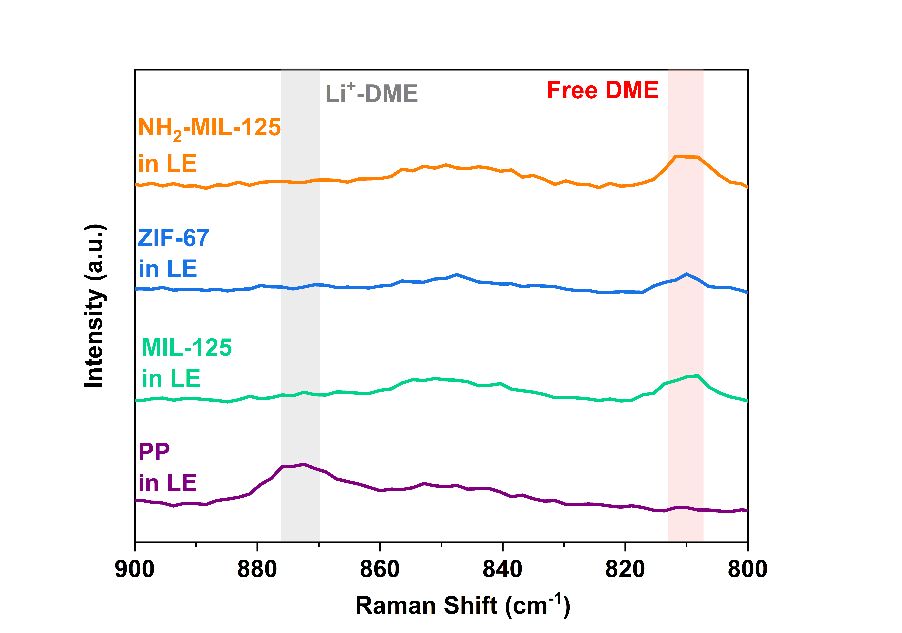


**Figure S19.** Raman spectra of liquid electrolyte on the PP, MIL-125, ZIF-67 and NH_2_-MIL-125 electrolytes.


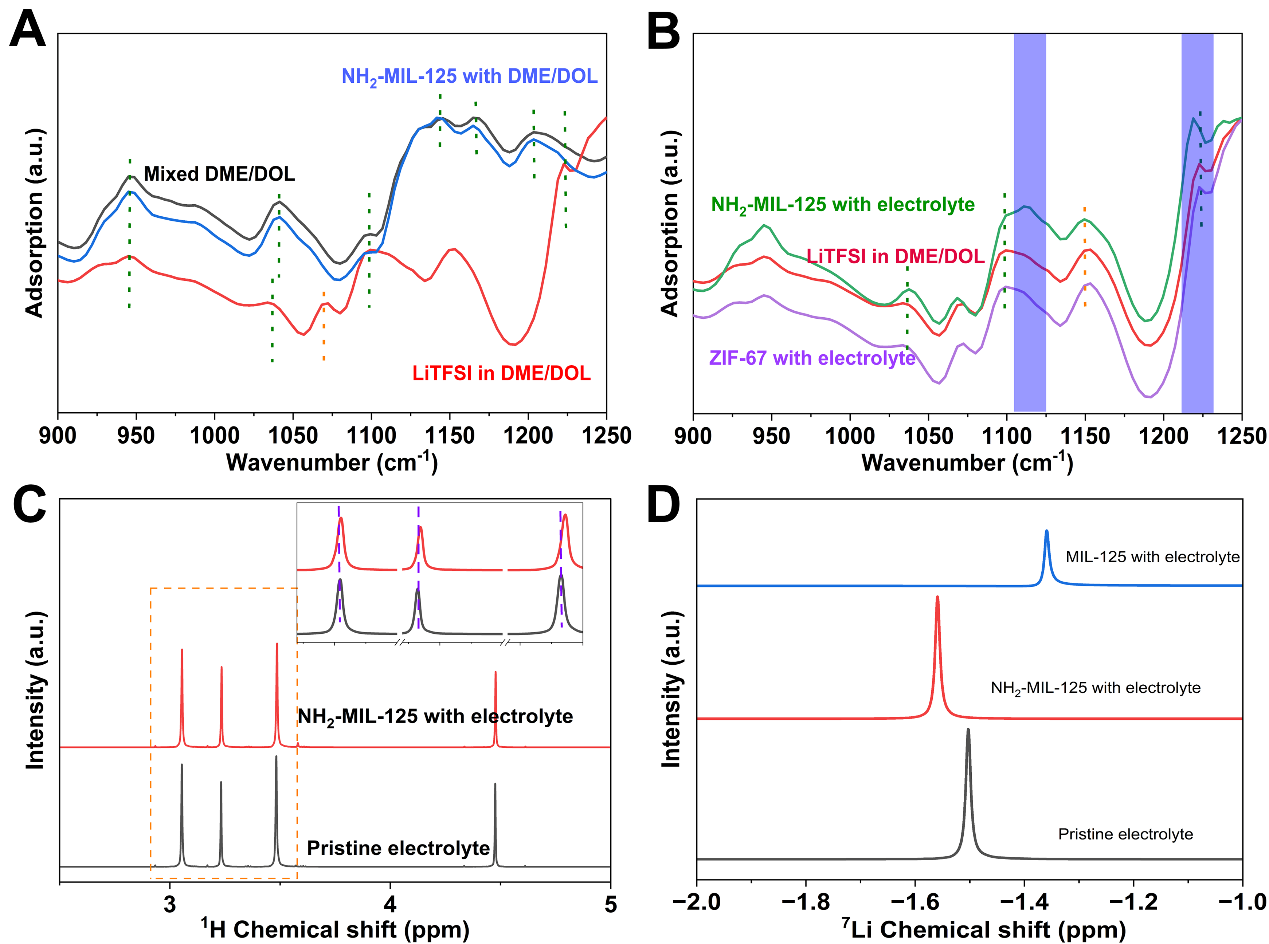


**Figure S20**. FT-IR spectra of (A) solvents and (B) electrolyte with/without MOFs. Comparisons of (C) H and (D) Li NMR with the introduction of NH_2_-MOFs.


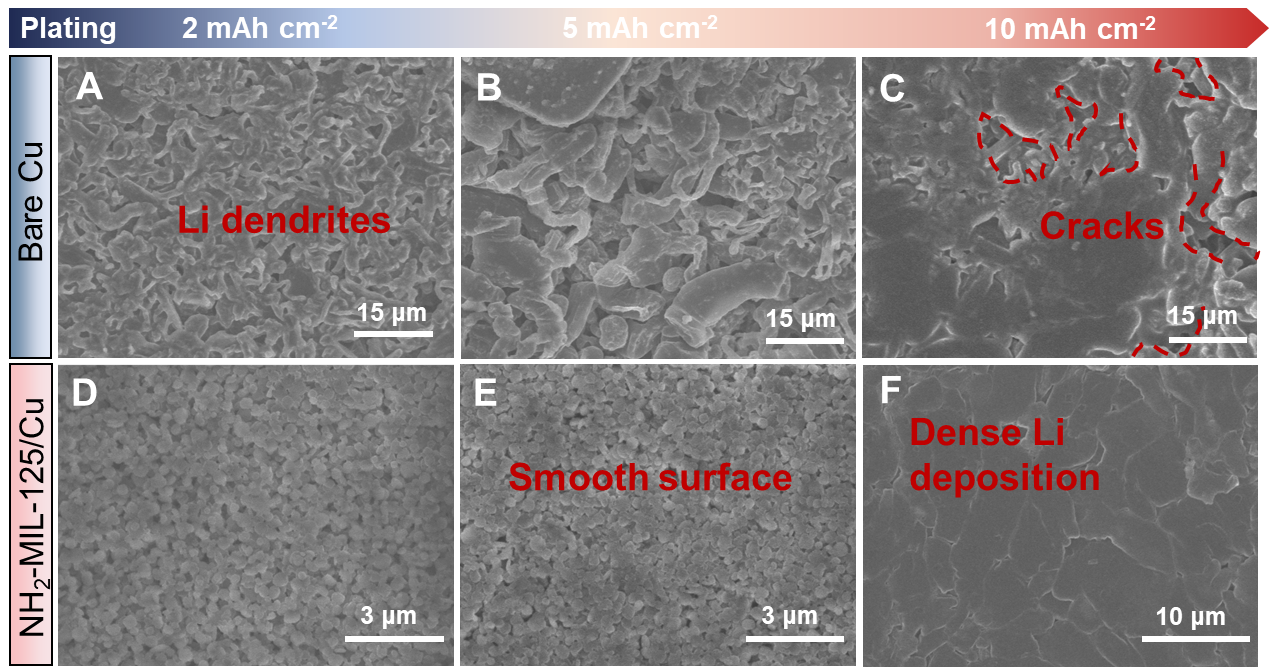


**Figure S21.** (A-C) Ex-situ SEM images of the bare Cu after plating 2 mAh cm^-2^, 5 mAh cm^-2^ and 10 mAh cm^-2^ of Li. (D-F) Ex-situ SEM images of the NH_2_-MIL-125/Cu after plating 2 mAh cm^-2^, 5 mAh cm^-2^ and 10 mAh cm^-2^ of Li.


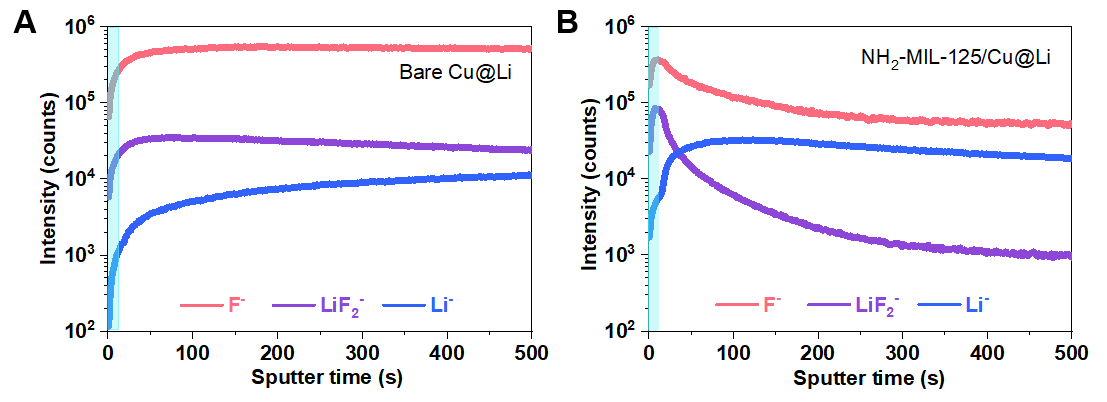


**Figure S22.** TOF-SIMS normalized depth profiling of F^−^, LiF_2_^−^, and Li^−^ fragments of cycled electrodes: (A) bare Cu@Li; (B) NH_2_-MIL-125/Cu@Li.

**
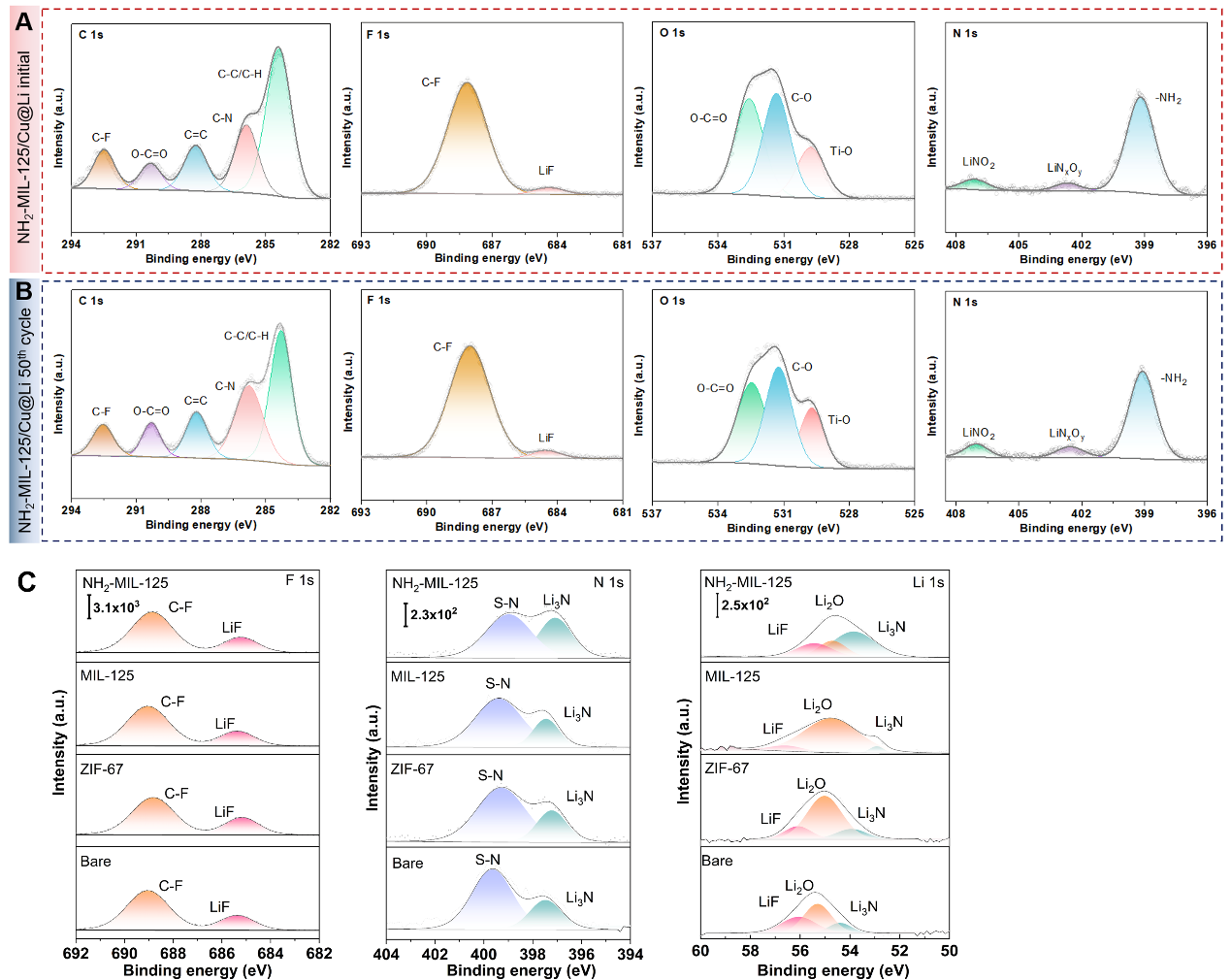
**

**Figure S23.** XPS spectra of C 1s, F 1s, O 1s, and N 1s for NH_2_-MIL-125/Cu@Li electrode (A) after the initial deposition and (B) after 50 cycles. Note: The 5 mAh cm^-2^ of Li amount was pre-deposited on the NH_2_-MIL-125/Cu before cycling. (C) The XPS spectra of F 1s, N 1s and Li 1s in the Cu electrodes without any Li pre-plating after 10 cycles.


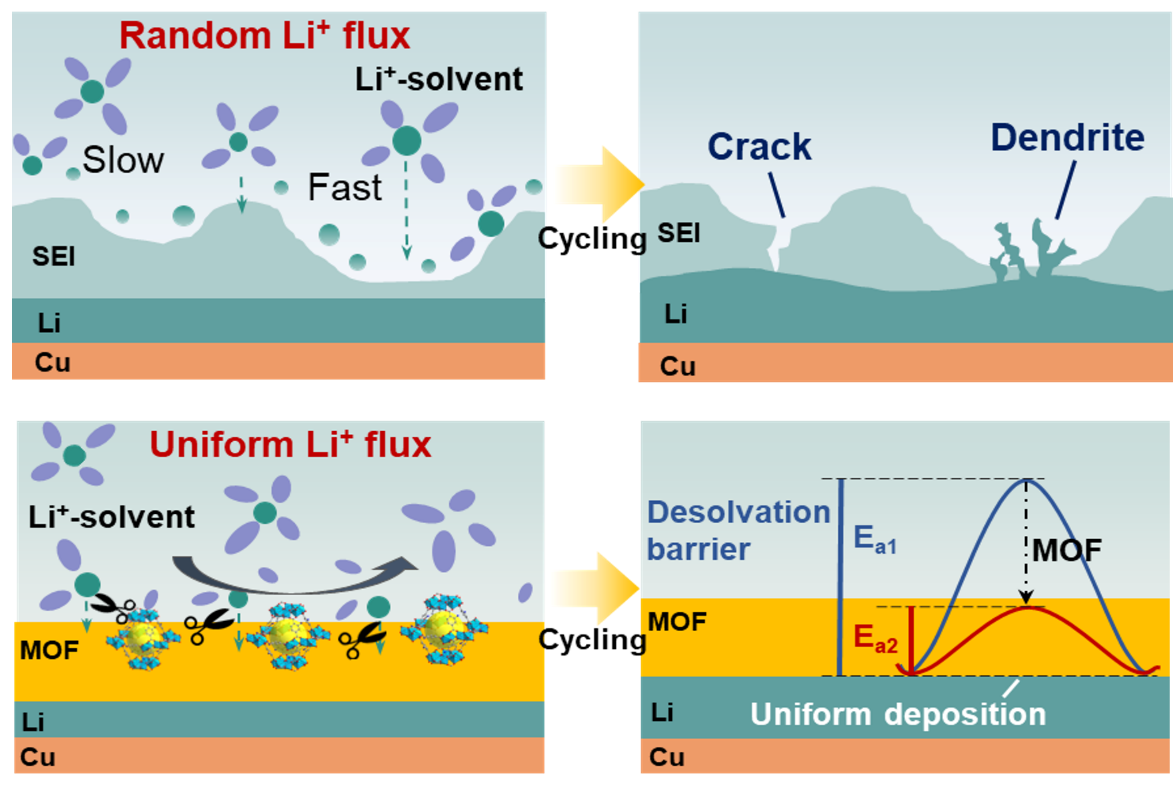


**Figure S24.** Schematic illustrations of desolvation behaviors catalyzed or sieved by MOF layer with pre-plating the Li capacity of 5 mAh cm^-2^.


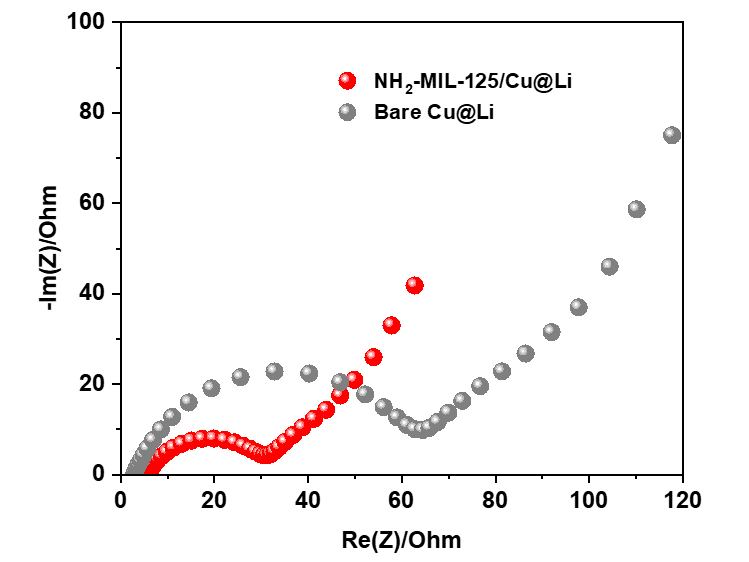


**Figure S25.** EIS comparison of the bare Cu@Li||LFP and NH_2_-MIL-125/Cu@Li||LFP full cells.


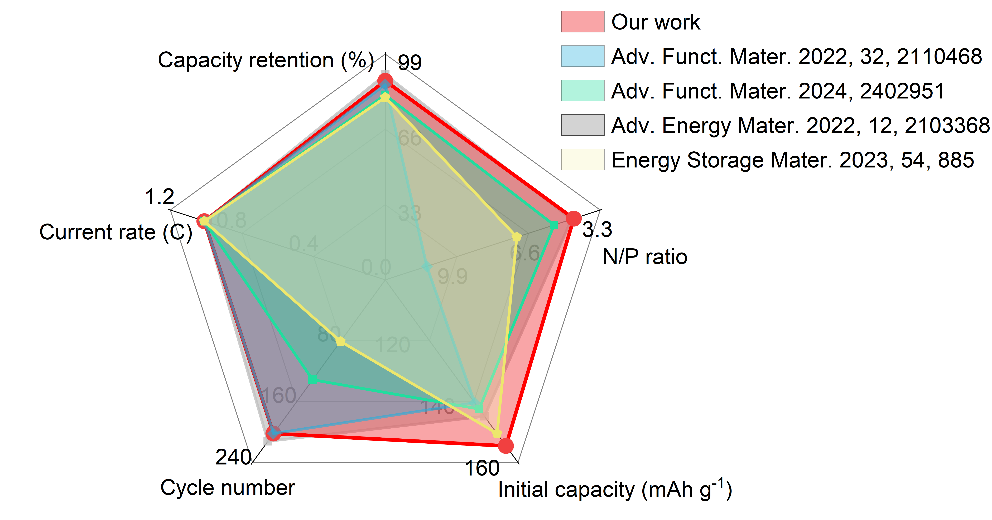


**Figure S26.** Comparison of capacity retention (%), N/P ratio, initial capacity (mAh g^-1^), cycle number and current rate (C) with previous studies.


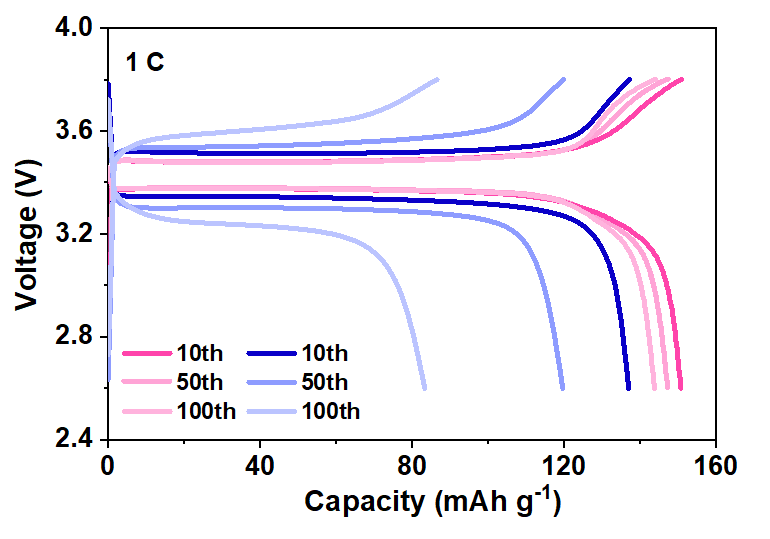


**Figure S27.** Selected discharge-charge curves of the NH_2_-MIL-125/Cu@Li||LFP cell at 1 C rate.

**
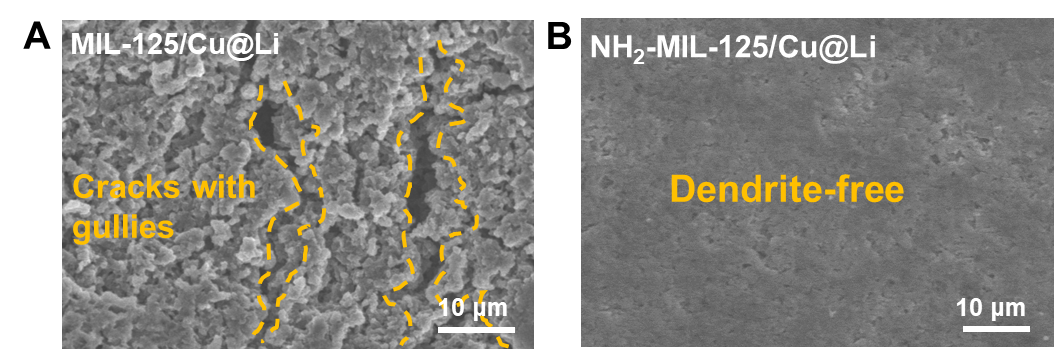
**

**Figure S28.** High-resolution SEM images of (A) MIL-125/Cu@Li and (B) NH_2_-MIL-125/Cu@Li in the cycled full battery under a low-temperature environment of 0 °C.

**
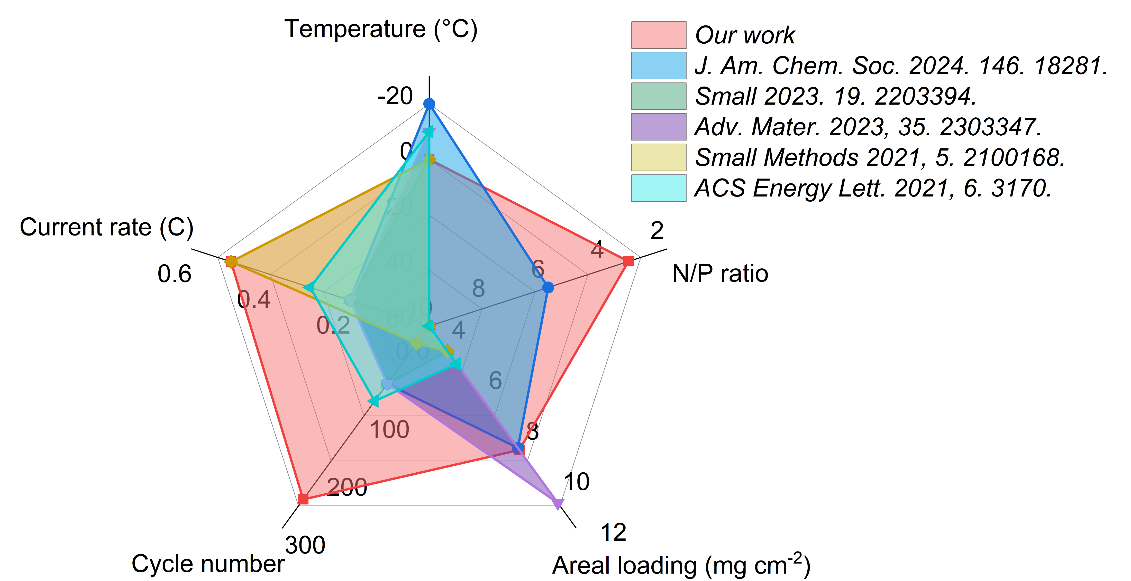
**

**Figure S29.** Comparison of operating temperature (°C), N/P ratio, cathode areal loading (mg cm^-2^), cycle numbers and current rate (C) with previous studies.


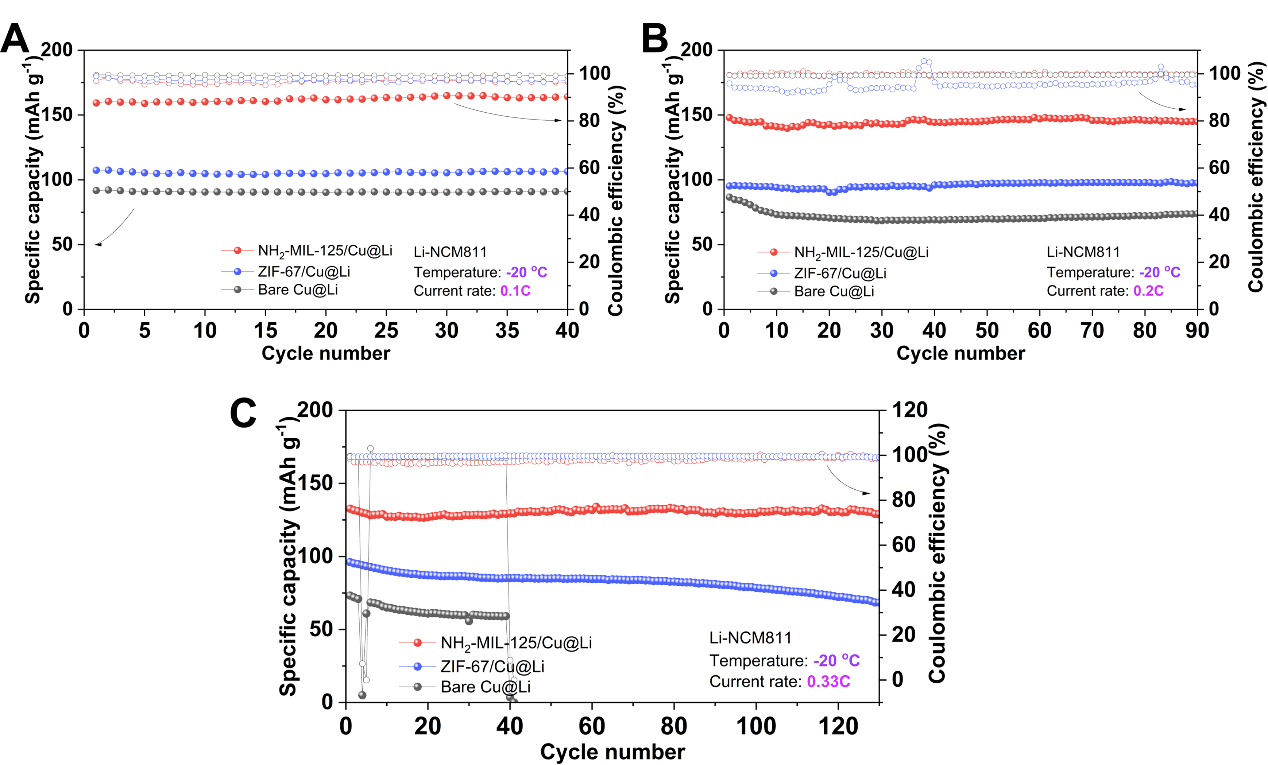


**Figure S30.** The performance of Li-NCM811 full cells cycled under -20 ^o^C at different current rates.

**Table S1.** Summary of lifespan for recently reported Cu electrodes under similar operating conditions.

| **Electrode** | **Current density (mA cm^-2^)** | **Cycle number (cycles)** | **Literatures** |
| --- | --- | --- | --- |
| MM@Cu | 1 | 270 | Adv. Funct. Mater, 2022, 32, 2206388. (Ref.S1) |
| S-CuO@Cu | 1 | 150 | Adv. Sci. 2023, 10, 2301288. (Ref.S2) |
| COFsLi-Cu | 1 | 300 | Energy Environ. Sci. 2024, 17, 1117. (Ref.S3) |
| Li/COF-Cu | 1 | 360 | Adv. Mater. 2024, 36, 2313076. (Ref.S4) |
| PPy-NS | 1 | 200 | ACS Sustainable Chem. Eng. 2021, 9, 2543. (Ref.S5) |
| Mo_2_N@CNF | 1 | 150 | Adv. Mater. 2019, 31, 1904537. (Ref.S6) |
| NCRA | 1 | 120 | Chem. Eng. J. 2019, 363, 270. (Ref.S7) |
| 3D CuZn | 1 | 150 | ACS Energy Lett. 2019, 5, 180. (Ref.S8) |
| SiO_2_/ERG-CNF | 1 | 200 | Adv. Energy Mater. 2018, 8, 1800564. (Ref.S9) |
| VA-CuO-Cu | 1 | 200 | Adv. Energy Mater. 2018, 8, 1703404. (Ref.S10) |
| Cu-CuO-Ni | 1 | 200 | Adv. Mater. 2018, 30, 1705830. (Ref.S11) |
| 3D Cu | 1 | 150 | Adv. Energy Mater. 2018, 8, 1800266. (Ref.S12) |
| HKUST-1@Cu | 1 | 300 | Angew. Chem. Int. Ed. 2022, 134, e202116291. (Ref.S13) |
| Co@N-G | 1 | 250 | Adv. Funct. Mater. 2020, 30, 2000786. (Ref.S14) |
| ZnO-CuZn mesh | 1 | 100 | Nano Lett. 2019, 19, 1832. (Ref.S15) |
| **NH_2_-MIL-125/Cu** | **1** | **400** | **Our work** |

**Table S2.** Summary of lifespan for recently reported Li electrodes under similar operating conditions.

| **Electrode** | **Current density (mA cm^-2^)** | **Lifespan (h)** | | **Literatures** |
| --- | --- | --- | --- | --- |
| Li-In | 0.5 | 230 | Energy Storage Mater. 2020. 33. 423. (Ref.S16) | |
| MASPLA-Li | 0.5 | 600 | Energy Storage Mater. 2022. 52. 210. (Ref.S17) | |
| PEGDA/LiDFOB-Li | 0.5 | 700 | Nano Energy 2022, 95, 106983. (Ref.S18) | |
| PA-MXene-Li | 0.5 | 900 | Adv. Mater. 2019, 31, 1901820. (Ref.S19) | |
| RIDAL-Li | 0.5 | 900 | Adv. Funct. Mater. 2022, 32, 2110468. (Ref.S20) | |
| MPTS-Li | 0.5 | 1000 | Adv. Funct. Mater. 2021, 31, 2104930. (Ref.S21) | |
| Li@Ti_3_C_2_T_x_/g-C_3_N_4_ | 0.5 | 1050 | Adv. Sci. 2022, 9, 2103930. (Ref.S22) | |
| SDMECO@HINC-Li | 0.5 | 1200 | Adv. Sci. 2022, 9, 2202244. (Ref.S23) | |
| MXene@CNF/Li | 0.5 | 1300 | Nano Energy 2020, 74, 104817. (Ref.S24) | |
| **NH_2_-MIL-125/Cu@Li** | **0.5** | **2000** | **Our work** | |

**Table S3.** Comprehensive comparison of low-temperature performance of Li-NCM811 batteries.

|  | **Electrode** | **Electrolyte** | **N / P ratio** | **Temperature (°C)** | **Current rate** | **Initial capacity (mAh g^-1^)** | **Lifespan** | **Capacity retention** | **Literature** |
| --- | --- | --- | --- | --- | --- | --- | --- | --- | --- |
| 1 | NCM811 | 1M LiClO_4_ in ES + 10 wt% FEC | 5 | -33 | 0.1 C | 152 | 100 cycles | 82 % | *Angew. Chem.* **2024**, *136*, e202310905. |
| 2 | NCM811  (2.8-4.8V) | 1 M LiFSI in EMC/FEC/DTF(1.5 : 1.5 : 7 by volume) | 191.4 | -40 | 0.2 C | 153.2 | 100 cycles | 93 % | *J. Am. Chem. Soc.* **2024**,*146*, 27644 |
| 3 | NCM811 | 0.5 M LiPF_6_ + 0.5 M LiTFSI+0.1 M LiNO_3_ in THF + 10 vol% FEC | 516 | -30 | 0.05 C | 152.6 | 50 cycles | 84.64 % | *Adv. Funct. Mater.* **2024**, *34*, 2309858. |
| 4 | NCM523 | 1 M LiTFSI in DOL/DME | 5.6 | -20 | 0.2 C | 115 | 200 cycles | > 100 % | *Energy Environ. Sci.* **2024**, *17*, 5468 |
| 5 | NCM811  (2.8-4.6V) | 1.2 M LiPF_6_ in EC/EMC (w/w, 3:7) + 2 wt% VC + 0.1 mg ml^-1^ TpTta | 283.8 | -20 | 0.33 C | 152 | 700 cycles | > 80 % | *Energy Environ. Sci.* **2024**, *17*, 2642 |
| 6 | NCM811  (40 μm Li foil) | 1M LiFSI in THF+ 0.5 wt% NaPFO | 112.4 | -60 | 0.1 C | 80 | 70 cycles | 72 % | *Energy Environ. Sci.* **2024**, 17, 4537 |
| 7 | NCM811 | 1 M LiTFSI in DFEC/DEC | 4 | -30 | 0.1 C | 93 | 50 cycles | 95 % | *Adv. Energy Mater.* **2021**, *11*, 2100935. |
| 8 | NCM811 | 4 M LiFSI in DOL/TTE(1:1 by volume) +2 wt.%LiNO_3_ | 681.6 | -40 | 0.1 C | 66 | 300 cycles | 95.44 % | *Adv. Energy Mater.* **2024** 2401961. |
| 9 | NCM811  (2.7-4.5V) (40 μm Li foil) | 0.8 M LiTFSI, 0.2 M LiDFOB in (SN/HFBA)(0.54:0.46) + 4 vol% FEC+1 mol% PEGDA+0.5 mol% AIBN | 51.6 | -10 | 0.1 C | 122 | 150 cycles | 85.3 % | *Adv. Mater.* **2024**, 2403191 |
| **10** | **NCM811** | **Commercial low-temperature electrolyte** | **3.3** | **-20** | **0.1C**  **0.2C**  **0.33C** | **159**  **148**  **133** | **40 cycles**  **90 cycles**  **130 cycles** | **103%**  **98.0%**  **97.0%** | **This work** |

**Reference**

[1] X. Shen, *et al.* Lithiophilic interphase porous buffer layer toward uniform nucleation in lithium metal anodes. *Adv. Funct. Mater.* **2022**, *32*, 2206388.

[2] Y. Liu, *et al.* Integrated gradient Cu current collector enables bottom-up Li growth for Li metal anodes: role of interfacial structure. *Adv. Sci.* **2023**, *10*, 2301288.

[3] L. Yue, *et al.* In-situ interface engineering of highly nitrogen-rich triazine-based covalent organic frameworks for ultra-stable, dendrite-free lithium-metal anode. *Energy Environ. Sci*. **2024**, *17*, 1117.

[4] S. Zheng, *et al.* Three-dimensional crown ether covalent organic framework as interphase layer toward high-performance lithium metal batteries. *Adv. Mater.* **2024**, *36*, 2313076.

[5] W. Yao, *et al.* Polypyrrole nanotube sponge host for stable lithium-metal batteries under lean electrolyte conditions. *ACS Sustainable Chem. Eng*. **2021**, *9*, 2543-2551.

[6] L. Luo, *et al.* A 3D lithiophilic Mo_2_N-modified carbon nanofiber architecture for dendrite-free lithium-metal anodes in a full cell. *Adv. Mater.* **2019**, *31*, 1904537.

[7] L. Chen, *et al.* Self-supporting lithiophilic N-doped carbon rod array for dendrite-free lithium metal anode. *Chem. Eng. J.* **2019**, *363*, 270-277.

[8] D. Zhang, *et al.* Lithiophilic 3D porous CuZn current collector for stable lithium metal batteries. *ACS Energy Lett.* **2019**, *5,* 180-186

[9] Q. Song, *et al.* Vertically grown edge-rich graphene nanosheets for spatial control of Li nucleation. *Adv. Energy Mater.* **2018**, *8*, 1800564

Vertically grown edge-rich graphene nanosheets for spatial control of Li nucleation

[10] C. Zhang, *et al.* Vertically aligned lithiophilic CuO nanosheets on a Cu collector to stabilize lithium deposition for lithium metal batteries. *Adv. Energy Mater.* **2018**, *8*, 1703404.

[11] S. Wu, *et al.* Lithiophilic Cu-CuO-Ni hybrid structure: advanced current collectors toward stable lithium metal anodes. *Adv. Mater.* **2018**, *30*, 1705830.

[12] H. Zhao, *et al.* Compact 3D copper with uniform porous structure derived by electrochemical dealloying as dendrite-free lithium metal anode current collector. *Adv. Energy Mater.* **2018**, *8*, 1800266.

[13] Y. Ma, *et al.* A “blockchain” synergy in conductive polymer-filled metal-organic frameworks for dendrite-free Li plating/stripping with high coulombic efficiency. *Angew. Chem. Int. Ed*. **2022**, *134*, e202116291.

[14] T. Wang, *et al.* Regulating uniform Li plating/stripping via dual-conductive metal-organic frameworks for high-rate lithium metal batteries. *Adv. Funct. Mater.* **2020**, *30*, 2000786.

[15] S. Huang, *et al.* Chemical energy release driven lithiophilic layer on 1 m^2^ commercial brass mesh toward highly stable lithium metal batteries. *Nano Lett.* **2019**, *19*, 1832-1837.

[16] S. Liu, *et al.* Inducing uniform lithium nucleation by integrated lithium-rich li-in anode with lithiophilic 3D framework. *Energy Storage Mater*. **2020**. *33*, 423-431.

[17] J. Wang, *et al.* Hydrophobic lithium diffusion-accelerating layers enables long-life moisture-resistant metallic lithium anodes in practical harsh environments. *Energy Storage Mater*. **2022**, *52*, 210-219.

[18] W. Cao, *et al.* Organic-inorganic composite SEI for a stable Li metal anode by in-situ polymerization. *Nano Energy*, **2022**, *95*, 106983.

[19] D. Zhang, *et al.* Horizontal growth of lithium on parallelly aligned MXene layers towards dendrite‐free metallic lithium anodes. *Adv. Mater.* **2019**, *31*, 1901820.

[20] J. Wang, *et al.* Construction of moisture-stable lithium diffusion-controlling layer toward high performance dendrite-free lithium anode. *Adv. Funct. Mater.* **2022**, *32*, 2110468.

[21] Z. Wen, *et al.* Anticorrosive copper current collector passivated by self-assembled porous membrane for highly stable lithium metal batteries. *Adv. Funct. Mater.* **2021**, *31*, 2104930.

[22] F. Zhao, *et al.* Constructing artificial sei layer on lithiophilic MXene surface for high-performance lithium metal anodes. *Adv. Sci.* **2022**, *9*, 2103930.

[23] J. Zhang, *et al.* Tuning 4f-center electron structure by Schottky defects for catalyzing Li diffusion to achieve long-term dendrite-free lithium metal battery. *Adv. Sci.* **2022**, *9*, 2202244.

[24] C. Wang, *et al.* Topological design of ultrastrong MXene paper hosted Li enables ultrathin and fully flexible lithium metal batteries. *Nano Energy* **2020**, *74*, 104817.
